# Cooperative antitumor activities of carnosic acid and Trastuzumab in ERBB2^+^ breast cancer cells

**DOI:** 10.1186/s13046-017-0615-0

**Published:** 2017-11-03

**Authors:** Carolina D’Alesio, Grazia Bellese, Maria Cristina Gagliani, Cinzia Aiello, Elena Grasselli, Gianluca Marcocci, Angela Bisio, Sara Tavella, Tiziana Daniele, Katia Cortese, Patrizio Castagnola

**Affiliations:** 1 DIMES, Department of Experimental Medicine, Human Anatomy, University of Genoa, Via Antonio de Toni 14, 16132 Genoa, Italy; 2 DiMI, Department of Internal Medicine and Medical Specialities, University of Genoa, Viale Benedetto XV 2, 16132 Genoa, Italy; 3 Department of Integrated Oncological Therapies, IRCCS AOU - San Martino – IST, Largo Rosanna Benzi 10, 16132 Genoa, Italy; 4 DISTAV, Department of Earth, Environment and Life science, University of Genoa, Corso Europa 26, 16132 Genoa, Italy; 5 DIFAR, Department of Pharmacy, University of Genoa, Via Brigata Salerno 13, 16147 Genoa, Italy; 6 San Raffaele Scientific Institute, Experimental Imaging Centre, Via Olgettina 60, 20132 Milan, Italy

**Keywords:** Breast cancer, Carnosic acid, Trastuzumab, ERBB2, Cell cycle, Migration, Autophagy, Lysosomes, Transmission electron microscopy

## Abstract

**Background:**

ERBB2 is overexpressed in up to 20–30% of human breast cancers (BCs), and it is associated with aggressive disease. Trastuzumab (Tz), a humanized monoclonal antibody, improves the prognosis associated with ERBB2-amplified BCs. However, the development of resistance remains a significant challenge. Carnosic acid (CA) is a diterpene found in rosemary and sage, endowed with anticancer properties. In this in vitro study, we have investigated whether Tz and CA have cooperative effects on cell survival of ERBB2 overexpressing (ERBB2^+^) cells and whether CA might restore Tz sensitivity in Tz-resistant cells.

**Methods:**

We have studied BC cell migration and survival upon CA and Tz treatment. In particular, migration ability was assessed by transwell assay while cell survival was assessed by MTT assay. In addition, we have performed cell cycle and apoptosis analysis by high-resolution DNA flow cytometry and annexin-V, resazurin and sytox blue staining by flow cytometry, respectively. The expression of proteins involved in cell cycle progression, ERBB2 signaling pathway, and autophagy was evaluated by immunoblot and immunofluorescence analysis. Cellular structures relevant to the endosome/lysosome and autophagy pathways have been studied by immunofluorescence and transmission electron microscopy.

**Results:**

We report that, in ERBB2^+^ BC cells, CA reversibly enhances Tz inhibition of cell survival, cooperatively inhibits cell migration and induces cell cycle arrest in G0/G1. These events are accompanied by ERBB2 down-regulation, deregulation of the PI3K/AKT/mTOR signaling pathway and up-regulation of both CDKN1A/p21^WAF1^ and CDKN1B/p27^KIP1^. Furthermore, we have demonstrated that CA impairs late autophagy and causes derangement of the lysosomal compartment as shown by up-regulation of SQSTM1/p62 and ultrastructural analysis. Accordingly, we have found that CA restores, at least in part, sensitivity to Tz in SKBR-3 Tz-resistant cell line.

**Conclusions:**

Our data demonstrate the cooperation between CA and Tz in inhibiting cell migration and survival of ERBB2^+^ BC cells that warrant further studies to establish if CA or CA derivatives may be useful in vivo in the treatment of ERBB2^+^ cancers.

**Electronic supplementary material:**

The online version of this article (10.1186/s13046-017-0615-0) contains supplementary material, which is available to authorized users.

## Background

ERBB2 is a member of the epidermal growth factor (EGF) receptor (ERBB1) family and is chaperoned by HSP90; unlike other ERBB members (ERBB1, ERBB3 and ERBB4), ERBB2 has no soluble ligands [1]. ERBB2 is amplified and overexpressed in 20% to 30% of human breast cancers (BCs), and it is often associated with aggressive disease and poor prognosis [2, 3]. Trastuzumab (Tz, Herceptin®), a humanized monoclonal antibody [4], binds the extracellular region of ERBB2 and inhibits receptor signaling via several mechanisms, including the down-regulation of both the Ras/Raf/MAPK pathway, which contributes to cell proliferation [5], and the PI3K/AKT pathway, which regulates cell survival [6, 7]. Moreover, nuclear accumulation of the cell cycle inhibitor CDKN1B/p27^KIP1^, and cell cycle arrest have been reported [8, 9]. The introduction of Tz therapy and, more recently, of the first successful HER2-targeted antibody–drug conjugate (ADC), trastuzumab emtansine (T-DM1; Kadcyla; Genentech) have improved significantly progression free survival and overall survival in ERBB2-amplified metastatic BCs [10]. Nevertheless, the presence of primary and acquired resistance, as well as serious adverse side effects to Tz and T-DM1 treatments remain a significant common challenge [11, 12]. The catabolic process of autophagy is considered to be associated with resistance to chemotherapy and targeted inhibitors in cancers [13]. Indeed, autophagy induced by anti-ERBB2 targeting agents like Tz and Lapatinib may allow cancer cells to survive the stress induced by the therapy [14, 15]. Thus, the inhibition of autophagy after treatment with Tz resulted in apoptotic cell death [16].

Rosemary (*Rosmarinus officinalis*) and common sage (*Salvia officinalis*) contain multiple bioactive components including carnosic acid (CA), rosmarinic acid, carnosol, caffeic acid, and ursolic acid, which have antioxidant, antinflammatory, anti-steatosis and anticancer properties [17–19]. Whole extracts and/or purified components isolated from rosemary have been shown to inhibit the in vitro and in vivo cell growth from solid tumors including BCs and leukemia cells [17]. The biological effects of CA on the modulation of cell cycle arrest, apoptosis and autophagy programs have been studied in different cancer cell types [17]. CA-mediated inhibition of the PI3K/AKT/mTOR pathway and induction of autophagy have been observed in hepatocarcinoma cells [20]. At difference, in myeloid leukemia cells the inhibition of AKT signaling has induced p27^KIP1^ expression and a block in the G1 phase of the cell cycle [20]. In colon adenocarcinoma cells, CA reduced leptin signaling through dephosphorylation of AKT, ERK and Insulin-like growth factor-I receptor, which have caused a reduced expression of both BCL-XL and Cyclin D1 resulting in cell cycle arrest [21]. Induction of apoptosis through the production of reactive oxygen species (ROS) or mediated by the AKT/IKK/NF-kB axis has been reported in different carcinoma cell lines [17, 22]. In contrast, in human glioblastoma (GBM) cell lines CA did not down-regulate the PI3K/AKT pathway, whereas it induced proteasomal down-regulation of Retinoblastoma, Cyclin B1, SOX2 and GFAP and cell cycle arrest with only a minor induction of apoptosis [23]. Therefore, these evidences corroborate the notion that CA does not elicit a common response but down-modulates distinct cellular pathways depending on a cancer cell-type specific context. In particular, in BC cell lines, a previous study showed that CA inhibited cell growth and suggested that ERBB2 was required for this activity [24]. Others reported that rosemary extract enhanced Tz effects on survival inhibition [25]. However, neither the active compound nor the molecular mechanisms at the basis of this interaction were provided.

In the present study, we aimed at investigating the mechanisms of action and the potential benefit of the combined treatment of CA and Tz in vitro in ERBB2^+^ BC cell lines SKBR-3 and BT474, which are sensitive to Tz, and in Tz-resistant SKBR-3 cells. We have found that CA exerts a reversible cooperation with Tz in boosting the inhibition of cell survival and cell migration, by inducing cell cycle arrest in G0/G1 phase. These events are coupled with the down-regulation of PI3K/AKT/mTOR pathway, the inhibition of late autophagy and derangement of the endolysosomal compartment. In addition, we have shown for the first time that co-treatment with CA and Tz partially restored the sensitivity to Tz in Tz-resistant SKBR-3 BC cells. These results suggest that a combined therapy of CA and Tz could represent a new and potentially less toxic approach worth of further investigations for the treatment of ERBB2^+^ BCs.

## Methods

### Plant material

Fresh aerial parts of *Salvia somalensis* Vatke were obtained from Centro Regionale di Sperimentazione ed Assistenza Agricola (Albenga, Italy). All reagents were analytical or high performance liquid chromatography (HPLC) grade. The isolation of the leaf constituents of *Salvia somalensis* (1.15 Kg) was performed following a procedure previously described [26]. Carnosic acid (CA), m.p. 190–192 °C was identified by comparison of its physical and spectroscopic data with those published in the literature [27] and obtained with a HPLC purity of 95%.

### Cell culture and drug treatments

BC cell lines SKBR-3, BT474, MCF7 and MDA-MB-231 were obtained from Banca Biologica and Cell Factory in IRCCS AOU San Martino – IST belonging to the European Culture Collection’s Organization. Tz resistant SKBR-3 (Tz-Res SKBR-3) cells were generated by continuous treatment of SKBR-3 cells with Tz 200 μg/ml for 10 months. MCF10A cells were obtained from NIH Institute and cultured according to the manufacturer’s instructions.

BC cells were cultured in complete medium (DMEM high glucose supplemented with 10% heat-inactivated fetal bovine serum, 1% glutamine, penicillin and streptomycin (Euroclone S.p.A., Milan, Italy). Tz (Genentech-Roche, South San Francisco, CA, USA) was donated by the UFA-Unità Farmaci Antiblastici of the IRCCS AOU - San Martino - IST. Tz was used at a concentration of 10 μg/ml for SKBR-3 (parental and Tz-resistant), MDA-MB-231 and MCF7 cells and at 0.24 μg/ml for BT474, respectively. CA was used at 27.5 μM for SKBR-3 (parental and Tz-resistant), MDA-MB-231 and MCF7 cells and 37.5 μM for BT474, respectively. Control cultures were challenged with DMSO (CA solvent) and human IgGs. Similarly, CA treated culture were also exposed to human IgGs and Tz treated cultures to DMSO, respectively.

### Cell survival assay

All BC cells were plated in 24-well plates in complete medium (triplicate of SKBR-3, BT474 and MDA-MB-231 28,000 cells/well, MCF7 15,000 cells/well) and CA and/or Tz were administered every 48 h for up to 7 or 10 days (d) as indicated. Cell survival was measured at different time points using the 3-(4,5-dimethylthiazol-2yl)-2,5-diphenyltetrazolium bromide (MTT) or Alamar Blue (Thermo Fisher Scientific, Waltham, MA, USA) colorimetric assay as described before [28], and as indicated in the manufacturer’s instructions, respectively.

### Cell migration assay

Cell migration assay was performed in quadruplicate using 8.0 μm pore size inserts in 24-well plates (BD Bioscience, Franklin Lakes, NJ). Fifty thousands MDA-MB-231 cells and 150,000 BT474 cells were seeded in the upper chamber and overnight starved (DMEM supplemented with 1% glutamine, penicillin and streptomycin). The day after, starvation medium was replaced, in the lower chamber, with complete medium supplemented with CA for MDA-MB-231 cells or CA and/or Tz for BT474 cells. Migrated cells were stained with Crystal Violet after 48 h for MDA-MB-231 cells and after 7d for BT474 cells (treatments every 48 h). Cell migration was quantified using ImageJ [29] as previously described [30].

### Flow cytometry (FCM) analysis

BT474 and SKBR-3 cells were treated with CA and/or Tz for 48 h. Both adherent and floating cells were then collected and centrifuged at 980 g for 5 min. Cell cycle analysis was performed through evaluation of DNA content in cell nuclei stained with DAPI by high resolution DNA flow cytometry (hr DNA-FCM) using a CyFlow ML flow cytometer (Sysmex-Partec Inc., Lincolnshire, IL, USA) [31]. Metabolic active, apoptotic and necrotic cells were evaluated using a Cyan ADP flow cytometer (Beckman Coulter, Brea, CA, USA) and the Vybrant® Apoptosis Assay Kit (Thermo Fisher Scientific) with a minor modification as we used the nuclear staining fluorochrome sytox blue (Thermo Fisher Scientific) in place of the sytox green.

### Antibodies

All primary antibodies used are listed in Additional file 1: Table S1.

### Immunofluorescence analysis

SKBR-3, BT474 and MCF10A cells were treated for 7d with CA and/or Tz (treatments every 48 h), fixed in 3% paraformaldehyde (PFA) in phosphate-buffered saline (PBS) pH 7.4 and then quenched with 30 mM NH_4_Cl. Cell permeabilization was performed with Triton for the Ki-67 staining and with saponin for LAMP 1 and LAMP 2 staining. Alexa-conjugated secondary antibodies were from Thermo Fisher Scientific. Image deconvolution and acquisition was performed with an Axio Imager A2M microscope equipped with an Apotome module (Carl Zeiss, Jena, Germany).

### Immunoblot analysis

BC cells were lysed using lysis buffer (Hepes pH 7.4 20 mM, NaCl 150 mM, 10% Glycerol, 1% Triton X-100) with protease inhibitors cocktail Complete (Roche Applied Science, Penzberg, Germany) and sodium orthovanadate or Phostop (Roche). Proteins were resolved on SDS-polyacrylamide gel electrophoresis and blotted on nitrocellulose (Thermo Fisher Scientific) or PVDF (Merck Millipore, Darmstadt, Germany) membranes. Detection was performed with ECL Detection Reagent (GE Healthcare) according to manufacturer’s protocol. ECL signals were detected, recorded and measured by either the GS-800 Calibrated Imaging Densitometer and the Quantity One Software (BioRad, Hercules, CA) or the Li-Cor scanner and Image Studio software (LI-COR Biosciences Inc., Lincoln, NE, USA) or the Uvitec Cambridge gel doc system and software (Cambridge, UK).

### Transmission electron microscopy (TEM)

For electron microscopy analysis, SKBR-3 and BT474 cells were seeded on glass chamber slides (Lab-Tek 177,380, Nalge Nunc int., Rochester, NY, USA) and treated for 7d. After drug treatments, cells were processed for electron microscopy [32] and observed with a CM10 electron microscope (Philips, Eindhoven, The Netherlands). Digital images were taken with a Megaview 3 camera. Analysis of morphologically identified multivesicular bodies (MVBs), autolysosomes (AL), autophagic vesicle/lipid droplets (AV/LDs) and lipid droplets (LDs) diameters were assessed in 10 cells for each treatment. The diameter of each organelle was measured with iTEM software package (Olympus-SYS; Olympus Corporation, Shinjuku, Tokyo, Japan) and plotted as histograms.

### RNA extraction and real-time and quantitative PCR (RT qPCR)

RNA was isolated using the Trizol reagent (Thermo Fisher Scientific), cDNA was synthesized and real-time quantitative PCR (RT qPCR) was performed in quadruplicate using 1 × IQTM SybrGreen SuperMix and CFX apparatus (Biorad, Milan, Italy). The relative quantity of target mRNA was calculated by the comparative Cq method using glyceraldehyde 3-phosphate dehydrogenase (GAPDH) as housekeeping gene (GAPDH fwd: 5′-ACCCACTCCTCCACCTTTGACG-3′; GAPDH Rev. 5′- CTCTTGTGCTCTTGCTGGGGCTG-3′), and expressed as fold induction with respect to controls [33]. PLIN2 primer pairs (PLIN2 Fwd 5′- TGTGAGATGGCAGAGAACGGT-3′; PLIN2 Rev. 5′-CTGCTCACGAGCTGCATCATC-3′) were designed ad hoc starting from the coding sequences of *Homo sapiens* available on the GenBank database (https://www.ncbi.nlm.nih.gov/genbank/) and synthesized by Tib MolBiol s.r.l. (Genova, Italy). PLIN1 primer pairs were purchased from Biorad (# qHsaCID0011127). Amplification conditions consisted of 2 min at 95 °C followed by 5 s at 95 °C and 30 s for PLIN1 or 45 s for PLIN2 at 60 °C for 40 cycles.

### Statistical analysis

All measurements here reported are presented as mean ± standard deviations. For MDA-MB-231 and MCF7 MTT assay, MDA-MB-231 cell migration assay and SKBR-3 and BT474 Alamar Blue cell survival assay we used a two-tailed distribution Student’s *t*-test. For ultrastructural studies, we used the Mann–Whitney test. For BT474 cell migration assay, cell cycle analyses and Tz-Res SKBR-3 MTT assay, we used one-way ANOVA plus post-hoc Tukey’s test. For the Ki-67 measurements we used one-way ANOVA plus post-hoc Bonferroni’s test. For SKBR-3 and BT474 MTT assays we used two-way ANOVA plus post-hoc Bonferroni’s test. For SKBR-3 and BT474 RT qPCR analysis we used one-way ANOVA Kruskal Wallis’s test plus the post-hoc Tukey’s test. Mean differences were considered statistically significant (*P* value) at *P* < 0.05.

## Results

### CA inhibits BC cell survival and migration and enhances Tz in vitro antitumor effects in ERBB2^+^ BC cells

To establish the in vitro antitumor effects of CA on BC cells and whether they depend on the expression of ERBB2, as previously suggested [24], we first analyzed ERBB2 negative cell lines. In particular, we selected MDA-MB-231 (estrogen and progesterone receptors negative) and MFC7 cells (estrogen and progesterone receptors positive) to study cell survival and migration, two relevant features associated with aggressiveness and metastatic potential. Cell viability was assessed by MTT assay up to 10 days (d). We demonstrate that CA at 27.5 μM, a concentration that we used previously in GBM cells [23], significantly decreased the survival of both MDA-MB-231 and MCF7 cells compared to control cells from 6 d of culture onward (Additional file 2: Figure S2A, B). To assess the migratory capability of MDA-MB-231 cells upon CA administration (MCF7 cells were unable to migrate in our experimental conditions as shown also by others [34]), we performed a transwell migration assay and found that CA drastically inhibited the migration of these cells compared to controls (Additional file 2: Figure S2C). Subsequently, we investigated the effects of CA in two ERBB2^+^ BC cell lines, the SKBR-3 (estrogen and progesterone receptors negative) and the BT474 cells (estrogen and progesterone receptors positive). In particular, we wondered whether CA could cooperate with Tz, a therapeutic antibody directed to ERBB2, to enhance the in vitro antitumor activities of this drug. To mimic a chronic drug administration, we first determined the concentrations of CA able to inhibit cell survival at 50% of the control in 7d of culture. These were 27.5 and 37.5 μM for CA in SKBR-3 and BT474, respectively (data not shown). Similarly, we determined the Tz concentration inhibiting cell survival at 50% of the control in 7d of culture. These were 10 μg/ml and 0.24 μg/ml in SKBR-3 and BT474, respectively (data not shown). All subsequent experiments were performed with these CA and Tz concentrations.

**Fig. 1:**
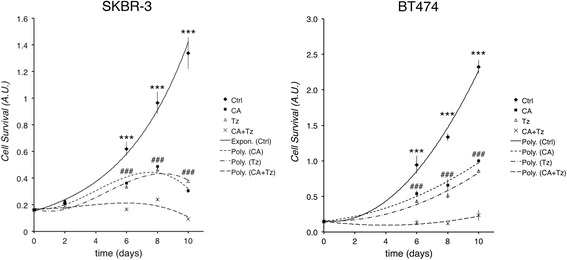
CA and Tz cooperatively inhibit SKBR-3 and BT474 *in vitro* cell survival. Cells were cultured for 10d with control medium (Ctrl), CA, Tz or CA + Tz supplemented medium, which was changed every 48 h. Cell survival is expressed as arbitrary units (A.U.) after exposure of MTT agent for 4 h. Exponential (Expon.) or polynomial (Poly.) interpolation curves are shown. Mean values and standard deviation (indicated as vertical bars) from three independent replicates (*n* = 3) are shown. Asterisks indicate statistical significativity *P* < 0.001 (***) of Ctr vs CA, Ctr vs Tz and Ctr vs CA + Tz. ### indicate statistical significativity *P* < 0.001 (^###^) of CA and Tz vs CA + Tz

We then performed the MTT assay up to 10 d to evaluate SKBR-3 and BT474 cell survival under CA, Tz and the combined treatment. As shown in Fig. 1, both CA and Tz significantly reduced BC cell survival in both cell lines from 6d onward (*P* < 0.01 at 6d, Ctr vs CA or Tz). Notably, the combined treatment strongly enhanced this reduction (*P* < 0.001 at 6d, Ctr vs CA + Tz, CA or Tz vs CA + Tz), suggesting a cooperative antitumor effect of the two drugs. To test whether the anti-survival effect of CA + Tz treatment was permanent or transient, we cultured SKBR-3 and BT474 cells with CA + Tz for 14 days. In parallel, we co-treated cells with CA + Tz for 6 d, then these cells were washed out and replaced with fresh control medium for additional 8 d. Cell survival analyses unveiled that both SKBR-3 and BT474 BC cells restarted proliferating when cultured in control medium, suggesting that the growth inhibitory effect was reversible (Additional file 3: Figure S3). We previously reported that CA inhibits the survival of both GBM cells and normal astrocytes [32]. To assess whether CA is effective in the inhibition of survival of a widely used normal mammary epithelial cell model and cooperates in that with Tz, we used the MCF10A cells [35, 36]. In particular, we treated these cells for 7d with the maximal concentration of CA and Tz used for the ERBB2^+^ cancer cell lines (37.5 μM CA and 10 μg/ml Tz, respectively) and performed the MTT assay. Our results showed, as expected, that CA inhibited MCF10A cell survival whereas Tz had no major effect on these cells (Additional file 4: Figure S4A).

**Fig. 2:**
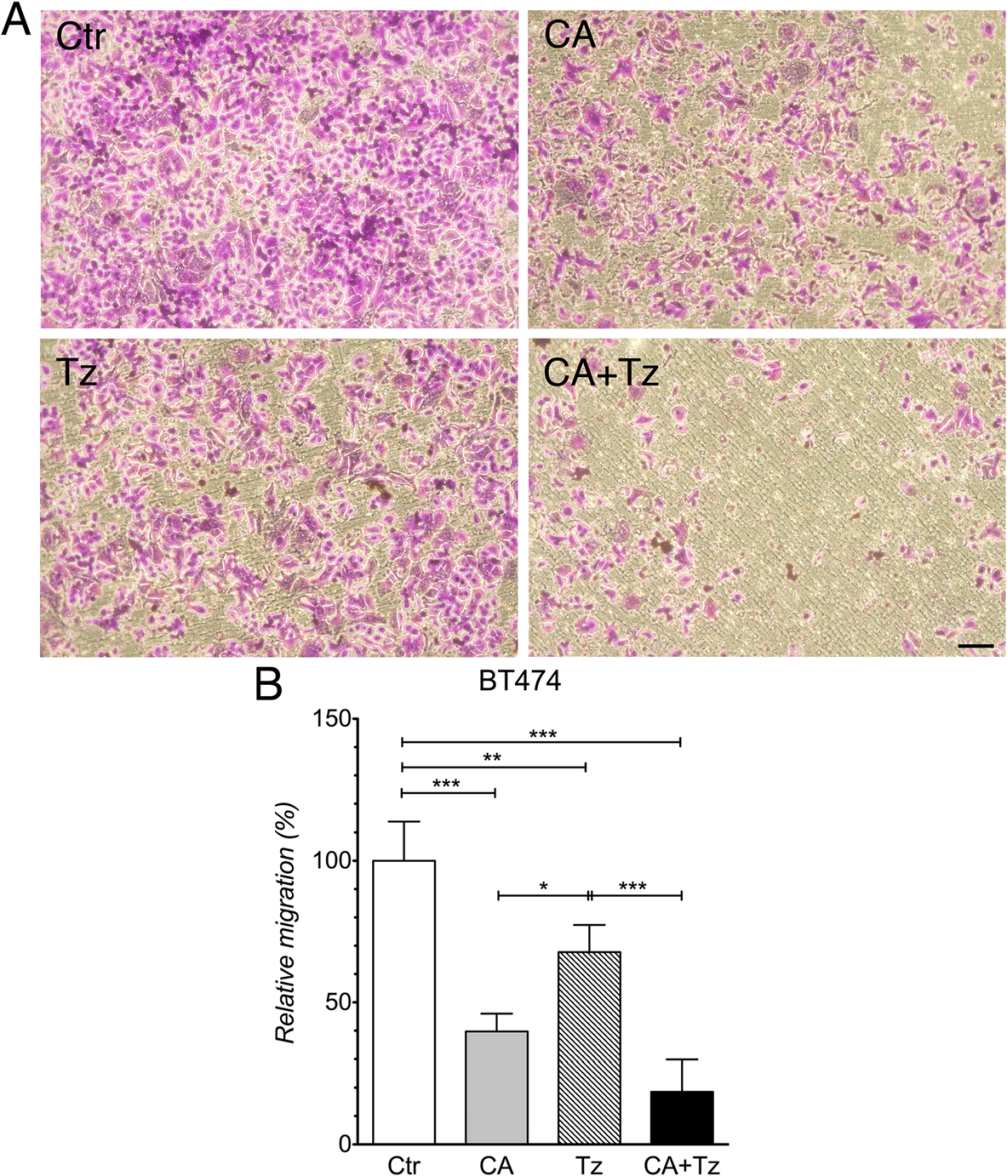
CA and Tz cooperatively inhibit in vitro BT474 cell migration. Cells were cultured after 48 h of starvation for 7d with control medium (Ctr), CA, Tz or CA + Tz supplemented medium in the lower migration chamber, which was changed every other day. **a** Representative images of the lower side of the migration membranes stained with Crystal Violet. Bar = 100 μm. **b** Relative cell migration expressed as percentage (%) compared to Ctr cultures was determined as detailed in Methods section. Mean values and standard deviation (indicated as vertical bars) from four independent replicates (*n* = 4) are shown. *P* < 0.005 (*), *P* < 0.01 (**), *P* < 0.001 (***)

As for MDA-MB-231 cells, we analyzed the migratory ability of the BT474 cells (SKBR-3 cells were unable to migrate in our settings as shown also by others [34], data not shown). We demonstrated a statistically significant reduction of cell migration under CA or Tz when used as single treatment (Fig. 2a-b). Remarkably, the combined treatment potentiated the inhibition of cell migration.

### CA in combination with Tz induces cell cycle arrest in G0/G1 and reduces the ERBB2^+^ BC cells fraction in G2/M

CA-mediated apoptosis was described in prostate cancer cells by modulation of the AKT/IKK/NF-KB pathway [22]; however, only a moderate early apoptosis was detected in human GBM cells [23]. To investigate whether the reduction of cell survival upon CA and Tz administration in ERBB2^+^ cells was due to activation of the apoptotic program, we treated SKBR-3 and BT474 with CA, Tz and in combination for 48 h. Cells were then analyzed by a FCM apoptosis assay, which allowed the identification of early apoptotic, late apoptotic and necrotic cells (Additional file 5: Figure S5A). This analysis showed that CA, Tz and the combined treatment did not induce early/late apoptosis or necrosis. To confirm this result after a prolonged exposure to drug treatments, we performed western blot analysis at 7d for the cleaved Caspase-9, a well-known apoptotic marker. As shown in Additional file 5: Figure S5B, we could not show major modulation of cleaved Caspase-9 levels in treated samples compared to controls in both cell lines, ruling out apoptosis as a relevant cause of survival inhibition in our experimental conditions.

**Fig. 3:**
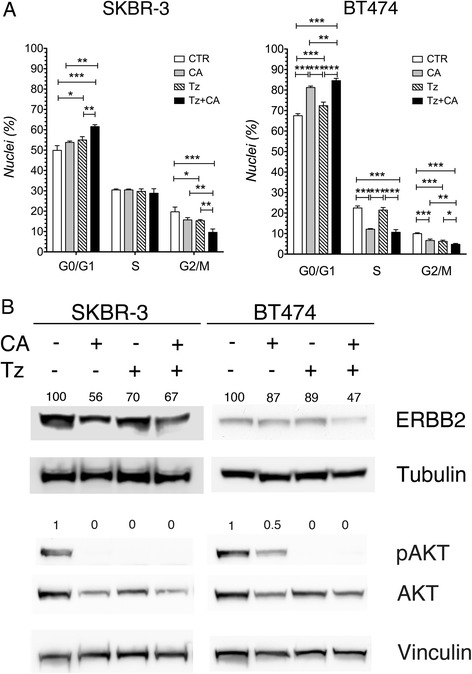
CA and Tz cooperatively arrest cell cycle in G0/G1 and down-regulate ERBB2 levels and signaling in SKBR-3 and BT474 cells. **a** High resolution DNA flow cytometric analysis of DAPI-stained nuclei of cells cultured for 48 h with control medium (Ctr), CA, Tz or CA + Tz supplemented medium. Both floating and adherent cells were collected for the analysis. Mean values and standard deviation (indicated as vertical bars) from four independent replicates (n = 4) are shown. *P* < 0.05 (*), *P* < 0.01 (**), *P* < 0.001 (***). **b** Cells were cultured for 7d with control medium (Ctr), CA, Tz or CA + Tz supplemented medium, which was changed every other day. A representative immunoblot analysis is shown, which was performed with anti-ERBB2, anti phospho-Ser^473^ AKT, anti AKT, anti-Tubulin and anti-Vinculin antibodies on whole cell lysates. Tubulin is shown as loading control for ERBB2. Vinculin is shown as loading control for pAKT and AKT. Numbers on each lane represent ERBB2 protein levels and pAKT/AKT level ratios determined as described in Methods

As we previously showed that CA induced cell cycle arrest in GBM cell [23], we examined whether CA inhibited cell cycle progression also in SKBR-3 and BT474 cells. Both cell lines were treated with CA, Tz and co-treated with CA + Tz for 48 h. The DNA content was evaluated by high resolution (hr) DNA-FCM (Fig. 3a) and representative DNA content histograms are shown in Additional file 6: Figure S6. This analysis showed that Tz-treated SKBR-3 and BT474 cultures accumulated cells in the G0/G1 phase of the cell cycle along with a reduction of cells in the G2/M phase compared to untreated cells. A statistically significantly increase of cells in the G0/G1 phase as well as a consistent decrease of CA and CA + Tz treated cells in the S phase was observed only in BT474 cells (Fig. 3a). Of note, in both cell types the co-treatment with CA + Tz increased the amount of cells in G0/G1 phase of the cell cycle with respect to single drug treatments, suggesting that CA cooperates with Tz in inducing a G0/G1 cell cycle arrest and in the reduction of the cell fraction in G2/M phase (Fig. 3a).

To clarify whether CA and Tz arrested cells in the G0 or in the G1 cell cycle phase, we further investigated the levels of the cell proliferation marker Ki-67, which only labels cells engaged in the cell cycle [37], upon CA, Tz or the combination CA + Tz in BT474 and SKBR-3 cells cultured for 7d (Additional file 7: Figure S7). Immunofluorescence analysis showed low-levels of Ki-67 expression in BT474 cells treated with CA, Tz and the combined treatment. At difference, SKBR-3 cells showed a significant decrease of Ki-67 only in Tz or CA + Tz treated cells (Additional file 7: Figure S7). Collectively, these data suggest that CA + Tz cells were mainly arrested in the G0 phase and exited the cell cycle before the accomplishment of the G1 phase [37].

### CA and Tz treatments do not cooperate in inducing endoplasmic reticulum and ROS stress in ERBB2^+^ BC cells

As in other cell types the mechanism of action of CA involves endoplasmic reticulum stress and ROS production [18], we investigated by immunoblot analysis the levels of HSP70 and Calreticulin, used as endoplasmic reticulum stress markers and Catalase, which is a ROS stress marker, in both BT474 and SKBR-3 cells treated with CA and Tz for 7d. This analysis, showed that CA and Tz modulates in a slightly different manner these stress markers in both cell types (Additional file 8: Figure S8). While Calreticulin was slightly induced by CA In SKBR-3, HSP70 levels showed a reduction upon CA, Tz and the combined treatment, compared to controls (Additional file 8: Figure S8). Finally, Catalase levels did not show major changes with both drugs, alone or in combination (Additional file 8: Figure S8). At variance, in BT474 cells both calreticulin and HSP70 showed an increment in CA and in CA + Tz treated cells compared to controls, while Catalase was induced by all treatments (Additional file 8: Figure S8). However, we did not find a cooperative CA and TZ inducing effect on the levels of these stress markers (Additional file 8: Figure S8).

### CA and Tz treatments inhibit the ERBB2 signaling pathway and cooperatively upregulate CDKN1B/p27^KIP1^ expression levels

It is well known that Tz inhibits ERBB2 downstream signaling pathways modulating AKT and MAPK signaling cascades [2, 38] and down-regulates ERBB2 expression [5, 39]. Reduced downstream signaling through these pathways activates p27^KIP1^ leading to G1 cell cycle arrest [40, 41]. Therefore, to investigate the mechanism/s through which CA and CA + Tz induce G0/G1 cell cycle arrest, we examined by immunoblot analysis ERBB2 protein levels and the phosphorylation status of AKT (after 7d of culture). This analysis showed that in SKBR-3 cells, ERBB2 levels were similarly reduced upon single or combined treatments (Fig. 3b). Notably, in BT474 cells, while the level of ERBB2 was only slightly down-regulated upon single treatments, the combination of both drugs induced a strong decrease of the receptor levels (Fig. 3b). The evaluation of ERBB2 downstream signaling revealed dephosphorylation of AKT Serine 473 upon single and combined treatments in both cell lines after 7d of culture (Fig. 3b).

**Fig. 4:**
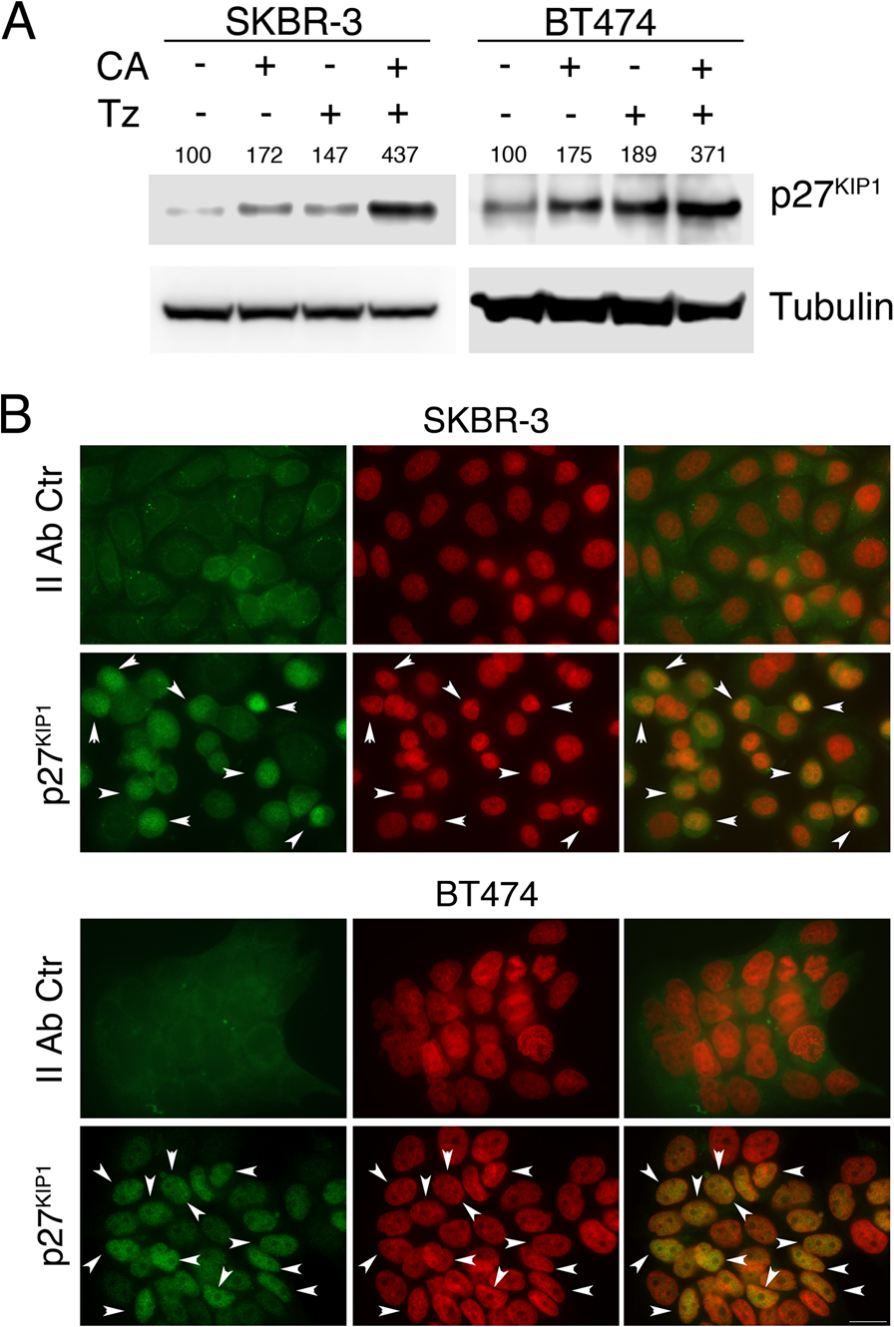
CA and Tz cooperatively upregulate p27^KIP1^ expression levels in SKBR-3 and BT474 cells. Cells were cultured for 7d with control medium (Ctr), CA, Tz or CA + Tz supplemented medium, which was changed every other day. **a** A representative immunoblot analysis is shown, which was performed with anti-p27^KIP1^ antibody on whole cell lysates. Tubulin is shown as loading control. Numbers on each lane represent p27^KIP1^ protein levels determined as described in Methods. **b** Epifluorescence images of SKBR-3 and BT474 cells cultured for 7d with CA + Tz fixed, permeabilized and challenged with anti p27^KIP1^ antibody and Alexa488-conjugated anti-mouse or with Alexa488-conjugated anti-mouse only as negative secondary antibody control (green signal). Nuclei were stained with DAPI (red signal). The extent of overlap is shown in yellow in the merged images (rightmost column) and arrowheads indicate some cells displaying p27^KIP1^ nuclear localization. Bar = 20 μm

We next evaluated the levels of two key regulators of the G1 cell cycle phase, CDKN1A/p21^WAF1^ and CDKN1B/p27^KIP1^ by immunoblot analysis. The results unveiled a strong activation of p27^KIP1^ upon single treatment and a synergic inducing effect upon combined treatment in both cell lines (Fig. 4a). A robust up-regulation of protein levels was also observed for p21^WAF1^ in CA, Tz and CA + Tz treated SKBR-3 and BT474 cells (Additional file 9: Figure S9). However, we could not detect a cooperative induction in the combined treatment (Additional file 9: Figure S9). As p27^KIP1^ is functional as a cyclin-dependent kinases (CDKs) inhibitor only when is located in the nucleus [42], we investigated its subcellular localization through immunofluorescence analysis in BT474 and SKBR-3 cells treated for 7d with CA + Tz. This analysis showed the presence of the protein p27^KIP1^ in the nuclei of CA + Tz ERBB2+ treated cancer cells (Fig. 4b). To establish whether CA induces CDK protein inhibitors in MCF10A cells we performed immunoblot analysis with p27^KIP1^ and p21^WAF1^ specific antibodies. We showed that, after 7d of drug treatments, CA dramatically increased the protein levels of the latter whereas reduced the former. On the contrary, a 7d treatment with Tz only slightly increased the levels of p27^KIP1^ with no major effect on those of p21^WAF1^ in these cells (Additional file 4: Figure S4B).

### CA inhibits late autophagy in ERBB2^+^ BC cells

**Fig. 5:**
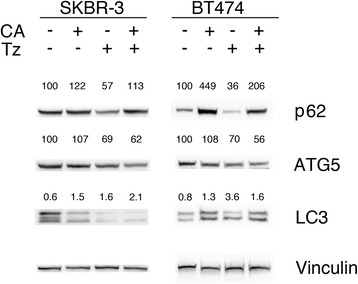
CA upregulates p62 in SKBR-3 and BT474 cells. Cells were cultured for 7d with control medium (Ctr), CA, Tz or CA + Tz supplemented medium, which was changed every other day. A representative immunoblot analysis is shown, which was performed with antibodies specific for p62, ATG5, LC3 and Vinculin on whole cell lysates. Vinculin is shown as loading controls. Numbers on each lane represent p62 and ATG5 levels and LC3 II/I level ratios, determined as described in Methods

Activation of autophagy in BC cells is a challenge regarding therapeutic resistance [15]. In this study, we assessed whether CA and Tz modulated autophagy in our cell model systems after 7d of exposure to treatments. To monitor the autophagy process, we evaluated both ATG5 and LC3 I/II levels by immunoblotting as functional reporters in SKBR-3 and BT474 cells treated with CA, Tz and CA + Tz for 7d (Fig. 5). Immunoblot results indicated that Tz led to increase of the LC3 II/I ratio and ATG5 down-regulation in both cell types. Similarly, CA caused an increase of the LC3 II/I ratio in both cell types whereas it did not modify ATG5 levels compared to controls. The combined treatment with CA and Tz appeared to be substantially in line with the effects of both drugs used as single treatments for what concerns the LC3 II/I ratio. With regards to ATG5 levels, the effects of CA + Tz were similar to those detected with the use of Tz alone (Fig. 5). As both the induction and suppression of autophagosome maturation may result in accumulation of LC3 II, to distinguish whether LC3 II accumulation was caused by autophagy induction or by inhibition of downstream steps, we used SQSTM1/p62 as a marker to measure the autophagic degradation activity [43]. The p62 protein specifically interacts with LC3 for its degradation by autophagolysosomes [43]. In fact, p62 accumulates when late autophagy is inhibited, and decreased levels can be observed when autophagy is induced [43]. We show that CA and the combined treatments CA + Tz induced accumulation of p62 in both SKBR-3 and BT474 cells, while Tz did not (Fig. 5). Interestingly, when we performed an immunoblot assay with the p62 antibody on MCF10A cells extracts, we detected higher levels of this protein in CA treated but not in Tz treated cells (Additional file 4: Figure S4B). The increase of LC3 II levels in ERBB2^+^ BC cells together with the increase of p62 suggests that CA is impairing the autophagic flux blocking a late maturation step of autophagosome degradation, i.e. the fusion to the lysosome or the lysosomal digestion.

**Fig. 6:**
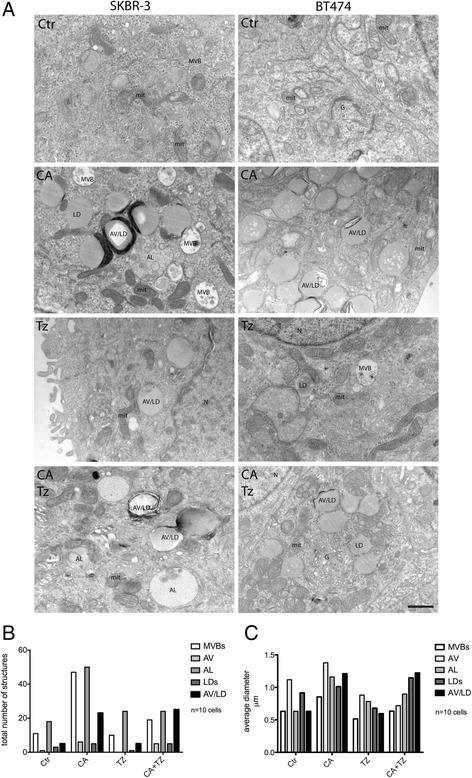
CA impairs autophagic flux accumulating autolysosomes (AL) and aberrant autophagic vesicles/lipid droplets (AV/LDs). **a** Representative TEM images of SKBR-3 and BT474 cells untreated (Ctr), CA-treated (+CA), Tz and CA + Tz treated for 7d. Four major categories of structures could be distinguished by morphological criteria in CA-treated cells: multivesicular bodies (MVBs), autolysosomes (AL) autophagic vesicle/lipid droplets (AV/LDs) and lipid droplets (LDs). Nuclei (N), Golgi (G), mitochondria (mit). Bar = 700 nm. **b**-**c** Histograms showing the number and diameter of each endocytic/autophagic structures upon different treatments in SKBR-3 cells. For this analysis, 10 whole cells were scored and measured for each category of structures with iTEM imaging software. Note that in CA-treated cells the most represented category is AV/LDs, followed by AL and MVBs

To better visualize cellular structures accumulated after 7d of CA, Tz and CA + Tz treatments, we performed TEM analysis in SKBR-3 cells. We quantified 4 categories of structures based on morphological criteria, the multivesicular bodies (MVB), autophagic vesicles (AV), autolysosomes (AL) and lipid droplets-derived autophagic vesicles (AV/LD). Interestingly, we found that CA induced a marked accumulation of MVB and enlarged AL, as well as lipid mobilization through the formation of aberrant AV/LD (Fig. 6a-c). These AV/LD structures often appear bi-phasic, with a low-density core surrounded by a more homogeneous dense lipid layer and by multilamellar tightly packed electron-dense membranes (Fig. 6a). In contrast, Tz-treated cells showed a decrease of AV and AL compared to CA-treated cells and very few AV/LD. Similarly to CA-treated cells, accumulation of AV/LD was observed in cells co-treated with CA + Tz, while the number of AL and MVBs was comparable to Tz-treated cells (Fig. 6b). Taken together, TEM analysis indicates that CA induces a late stage inhibition of autophagy by accumulating AL and aberrant AV/LD.

**Fig. 7:**
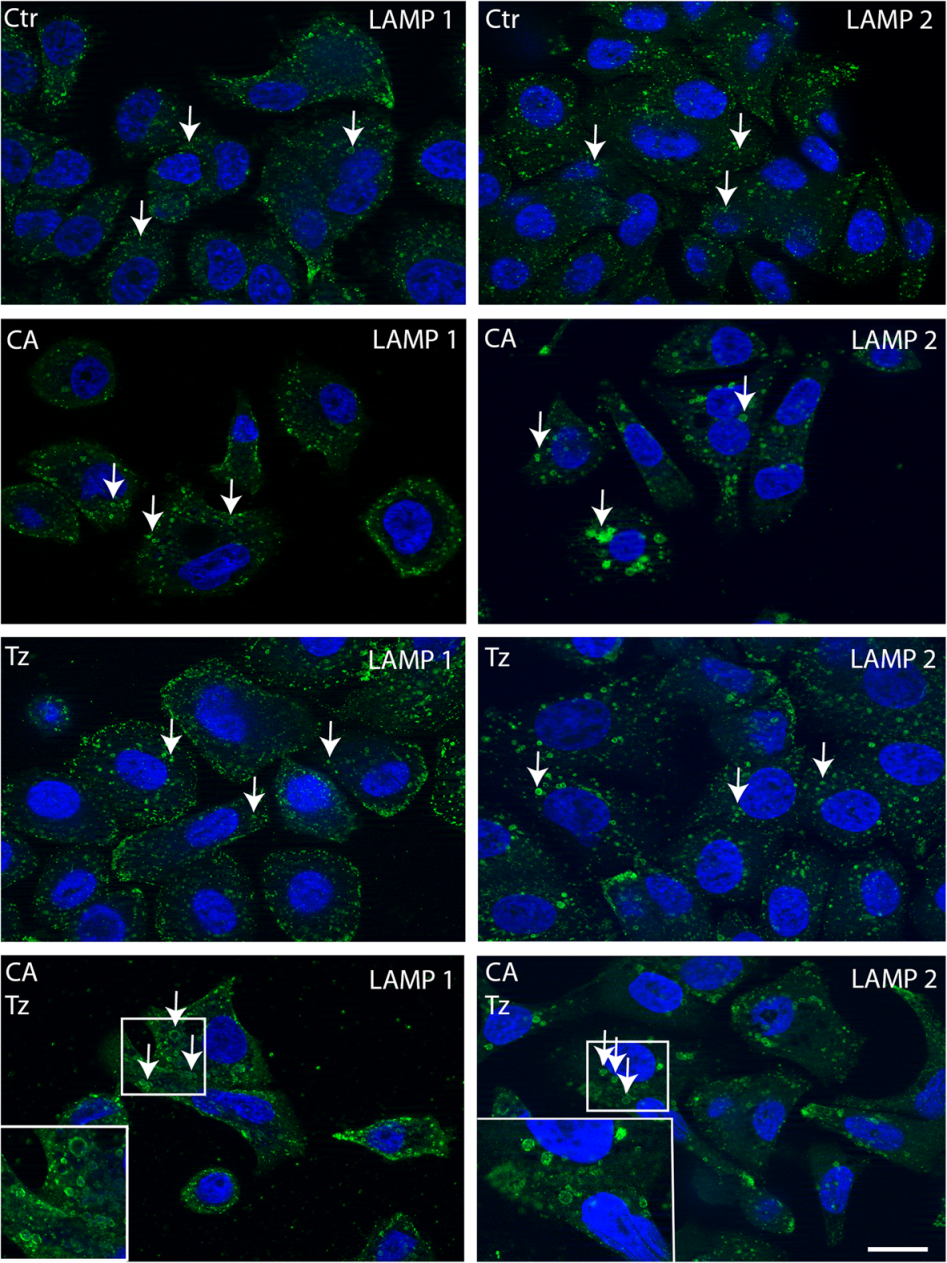
CA and CA + Tz dramatically affect lysosomal morphology in SKBR-3 cells. Representative confocal images of SKBR-3 cells cultured for 7d with CA, Tz and CA + Tz and untreated cells. Cells were fixed, permeabilized and challenged with anti-LAMP1 and anti-LAMP2 antibodies to detect lysosomes and Alexa488-conjugated anti-mouse secondary antibody (green signal). Nuclei were stained with DAPI (blue signal). Arrows indicates representative lysosomes in each condition. Of note, in CA and CA + Tz treated cells lysosomes (LAMP1 and LAMP2 positive) appear larger and clustered with respect to untreated cells (arrows). Bar = 20 μm. Insets represent a detail of the original image

Lysosomal protein degradation constitutes the final step of autophagy [44]. Accumulation of AL and AV/LD mediated by CA would reflect dramatic changes in the lysosomal compartment. We therefore assessed whether CA and CA + Tz affects the morphology of lysosomes as a result of the inhibition of the autophagic flux. LAMP1 and LAMP2, two glycoproteins abundantly expressed on the lysosomal membrane, are commonly used markers of lysosomes. Indeed, in SKBR-3 cells treated with CA and CA + Tz for 7d, the LAMP1 positive structures decreased but their size increased (Fig. 7), demonstrating that CA strongly impacts on lysosomal morphology. Accordingly, CA-induced alterations of the lysosomal compartment were also observed in MCF10A cells (Additional file 4: Figure S4C).

Previous reports showed that Tz induces lipid accumulation and differentiation in ERBB2^+^ BC cells [45]. To verify whether adipogenesis and/or differentiation were modulated by exposures to CA, Tz and to the combined treatment, we assessed by RT qPCR the transcription of Perilipin 1 (PLIN1) for adipogenesis and Perilipin 2 (PLIN2) for differentiation, two major genes that regulate lipid homeostasis in SKBR-3 and BT474 cells [46]. In addition, we also assessed the levels of PPARγ, the master regulator of adipocyte differentiation, by immunoblot analysis. We report that Tz, as well as CA + Tz induced a significant increase in PLIN1 mRNA levels in SKBR-3 cells while in BT474 PLIN1 mRNA levels were increased only by Tz (Additional file 10: Figure S10A). In contrast, CA did not up-regulated PLIN1 mRNA levels in both cell types. A significant up-regulation of PLIN2 expression levels was observed only in CA treated compared to Tz and CA + Tz treated SKBR-3 cells (Additional file 10: Figure S10A). Moreover, we did not observe modulation of PPARγ protein levels in SKBR-3 cells (Additional file 10: Figure S10B). Taken together, SKBR-3 cells appear more sensitive to CA-induced lipid mobilization compared to BT474 cells.

### CA partially restores Tz sensitivity in Tz-resistant SKBR-3 cells

**Fig. 8:**
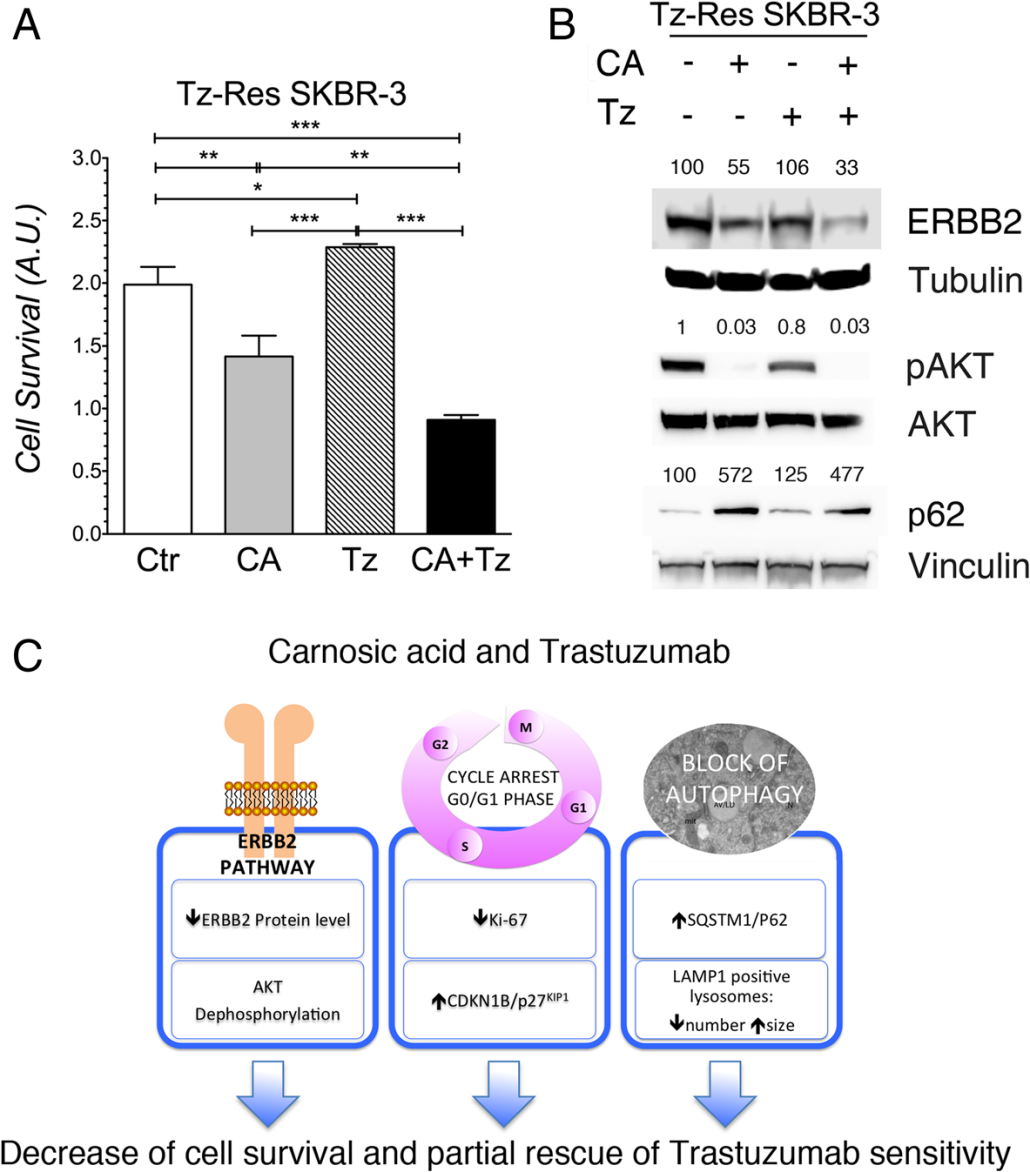
CA and Tz cooperatively inhibit in vitro survival of Tz-resistant SKBR-3 cells. **a** Cells were cultured for 7d with control medium (Ctr), CA, Tz or CA + Tz supplemented medium, which was changed every 48 h. Cell survival is expressed as arbitrary units (A.U.) after exposure of cultures to MTT for 4 h. Mean values and standard deviation (indicated as vertical bars) from three independent replicates (*n* = 3) are shown. *P* < 0.05 (*), *P* < 0.01 (**), *P* < 0.001 (***). **b** Cells were cultured for 7d with control medium (Ctr), CA, Tz or CA + Tz supplemented medium, which was changed every 48 h. A representative immunoblot analysis is shown, which was performed with anti-ERBB2, anti phospho-Ser^473^ AKT, anti AKT, anti p62, anti-Tubulin and anti-Vinculin antibodies on whole cell lysates. Tubulin is shown as loading controls for ERBB2. Vinculin is shown as loading controls for pAKT, AKT and p62. Numbers on each lane represent ERBB2 levels, pAKT/AKT ratios, and p62 levels determined as described in Methods. **c** Working model of CA and Tz effects in ERBB2+ breast cancer cells and proposed mechanisms of action

Primary or acquired resistance to Tz is still a major clinical problem for BC patients [5]. Indeed, to optimize drug response, Tz is usually used in combination with chemotherapeutic agents [5]. In this respect, we wondered whether CA could be active as anti-survival agent and rescue Tz response in Tz-resistant ERBB2^+^ BC cells. To test this hypothesis, we used SKBR-3 resistant cells (Tz-Res SKBR-3) able to growth in 200 μg/ml Tz, and determined cell survival through MTT assay. As for parental Tz-sensitive SKBR-3 cells, Tz-Res SKBR-3 cells were treated with CA (27.5 μM), Tz (10 μg/ml) or Tz + CA and after 7d of treatment, cell survival was measured (Fig. 8a). As expected, this analysis revealed that these cells were unresponsive to Tz but sensitive to CA. Remarkably, Tz-Res SKBR-3 cells treated with the combination Tz + CA showed a statistically significantly lower survival compared to that of cells treated with CA alone (Fig. 8a). To shed light on the mechanism through which Tz-Res SKBR-3 cells partially restored Tz sensitivity, we decided to examine the ERBB2 signaling pathway. Tz-Res SKBR-3 cells were treated with CA, Tz or CA + Tz for 7 d. Immunoblot analysis revealed that ERBB2 protein levels were decreased upon CA treatment and even more in CA + Tz treated cells whereas, as expected, ERBB2 levels were unchanged in cells treated with Tz alone (Fig. 8b). Concerning the ERBB2 downstream signaling, we observed in Tz-Res SKBR-3 a dramatic dephosphorylation of AKT Serine 473 upon CA and the combined treatment, while only a minor dephosphorylation was observed in cells treated with Tz alone (Fig. 8b). Interestingly, when we investigated the expression of p62 in Tz-Res SKBR-3, we observed that CA alone or in combination with Tz induced a dramatic up-regulation of p62 levels compared to Tz or controls (Fig. 8b).

## Discussion

The identification and characterization of the anticancer properties of natural products have aroused significant interest over the years. Several studies have shown that CA has antitumor properties in several cancer cell models [18]. However, a comprehensive evaluation of CA as anticancer agent in ERBB2^+^ BC cells is still lacking. For this reason, in this study we aimed at verifying whether CA was active in BC cells and specifically whether it could enhance the in vitro antitumor effects of Tz in ERBB2^+^ cells.

Differently from previous reports [24], our findings show that CA inhibits cell survival and migration in both ERBB2^−^ and ERBB2^+^ BC cells and, therefore, that its function is independent from the expression of this receptor. In addition, we have found that CA and Tz cooperate in inhibiting both migration and survival in ERBB2^+^ BC cells.

One of the major effects of both CA and Tz is to inhibit cell cycle progression [23, 47, 48]. Therefore, we have investigated cell distribution in cell cycle phases and demonstrated that the combined treatment with these two agents was more effective in blocking ERBB2^+^ BC cells in G0/G1 compared to CA and Tz alone. Accordingly, the decrease of Ki-67 expression, a cell proliferation marker, further showed that in ERBB2^+^ BC cells the block observed upon the Tz and CA + Tz treatments occurs mainly in G0. Noteworthy, others studies and ours have shown that CA causes a late G2 [23] or a G2/M block [47] in other cancer cell types. Thus, it appears that CA effects on cell cycle are cell context dependent. In ERBB2^+^ BC cells, our data strongly suggest that the cooperative mechanism exploited by CA and Tz causes cell cycle arrest in G0, which involves the induction of p27^KIP1^, as both drugs also cooperatively increase the levels of this CDKs inhibitor. According to this result, the observation of nuclear localization of p27^KIP1^ in ERBB2^+^ BC cells treated with CA + Tz is consistent with this proposed mechanism. However, we cannot rule out that the concomitant induction of p21^WAF1^, which we have also observed in GBM cells [23], may contribute, albeit to a lesser extent, to the cell cycle arrest determined by the CA + Tz treatment in these cells.

The chains of molecular events activated or repressed by CA and Tz, upstream to the induction of CDK inhibitors, remain unclear. In particular, we point to the evidence that although both ERBB2 expression levels and AKT phosphorylation levels were reduced by both drugs when used individually, we have not found a consistent combined effect on ERBB2 and AKT phosphorylation in both ERBB2^+^ BC cell lines. For example, CA and Tz cooperatively reduced ERBB2 levels in BT474 (estrogen receptor positive, ER^+^) but not in SKBR-3 (estrogen receptor negative, ER^−^) cells. As ER− status correlates with ERBB2 overexpression and poorly differentiated tumors [49], further studies are needed to determine if CA preferentially targets ER^+^ versus ER^−^ BC cells, therefore providing a conceivable explanation for the differences observed in the two ERBB2^+^ cell lines.

Cancer cells tend to activate autophagy for their survival. Here we have reported for the first time that CA inhibits autophagy by impairing the autophagic flux in a peculiar way that may impact cell survival of cells also exposed to Tz. Importantly, we demonstrate that CA does not affect ATG5, an E3 ubiquitin ligase necessary for the early stages of autophagosome formation, but that it increases p62 and LC3 II levels. These events likely lead to the accumulation of AL and aberrant AV/LD structures in ERBB2^+^ BC cells. In addition, we show that p62 induction by CA is more evident in SKBR-3 Tz-Res than in parental Tz-sensitive SKBR-3. Overall, our data demonstrate that CA disrupts the autophagic flux, thus impairing an important cancer survival pathway, which is also relevant in resistance to Tz. Notably, our data showing induction of p62 not only in ERBB2^+^ BC cells but also in MCF10A cells, strongly suggest that CA is a general inhibitor of autophagy.

Furthermore, we have shown that in Tz-resistant SKBR-3 cells CA and Tz cooperate in reducing ERBB2 levels along with a dramatic AKT dephosphorylation. Most importantly, we have proven a statistically significantly decrease of cell survival in Tz-resistant cells treated with CA + Tz compared to CA alone. This observation implies a partial rescue of the Tz action. It is tempting to speculate that the derangement of the endocytic/lysosomal pathway caused by the CA may impair the recycle of the ERBB2 to the plasma membrane. Certainly, further studies are needed to clarify the molecular mechanism responsible for the observed rescue of Tz activity.

As obstruction of the autophagic flux might lead to endoplasmic reticulum stress and, ultimately, apoptosis, we have consequently investigated apoptosis, necrosis, endoplasmic reticulum stress and ROS production as possible cellular processes that could impair cell survival in our ERBB2^+^ BC cell models (SKBR-3 and BT474). Though none of these processes were reported to have a major role in the Tz mechanism of action, several studies have highlighted their involvement in CA-induced inhibition of cell survival in cancer cell lines [18]. In our experimental context, CA and Tz, alone or in combination, have not elicited significant cell apoptosis or necrosis. However, the involvement of endoplasmic reticulum and ROS stress is less clear, since we have not found a consistent modulation of the HSP70 chaperone, calreticulin and catalase markers in the two ERBB2^+^ BC cell lines. Indeed, although CA and Tz up-regulated calreticulin, alone or in combination in both cell types, HSP70, which is also an endoplasmic reticulum stress marker, was up-regulated in BT474 but down-regulated in SKBR-3 cells.

Finally, we have evaluated whether an aberrant differentiation process could contribute to cell cycle arrest and survival inhibition in ERBB2^+^ BC cells treated with CA and Tz. An increase of neutral lipid production, associated with cell differentiation, was indeed reported to occur in SKBR-3 and in BT474 cells treated with Tz [45]. On the other hand, contrasting data exist on CA-mediated modulation of PPARγ, which is a key transcription factor up-regulated during adipogenesis [50, 51]. In this study, we have evaluated PPARγ protein levels, which is a major pro-adipogenic transcription factor [52], and the mRNA levels of PLIN1 and PLIN2, two proteins associated to the coat of lipid droplets that block lipolysis and increase during lipid accumulation [53–56], respectively. As we have observed that PPARγ levels were unchanged in SKBR-3 and that PLIN1 and PLIN2 mRNA levels were not modified consistently in the two ERBB2^+^ BC cells co-treated with CA and Tz, we conclude that the activation of an aberrant differentiation process, possibly activated by CA and Tz, is unlikely to have a major role in cell cycle arrest caused by CA and Tz in our experimental context.

Finally, we would point that CA inhibition of cell survival, the induction of both p21^WAF1^ and p62 and derangement of the endocytic/lysosomal pathway have been observed both in BC cells and in normal mammary epithelial cells. It should be noticed, however, that MCF10A cells are spontaneously immortalized mammary epithelial cells [35] with a doubling time that is actually shorter (16 h) [57] than that of the SKBR-3 and BT474 cells (44 and 72 h, respectively) [58, 59]. Therefore, it is very likely that MCF10A cells have a deranged control of the cell cycle perhaps explaining why we have observed p21^WAF1^ but not p27^KIP1^ induction mediated by CA.

Survival analysis in MCF10A treated with CA confirms and extends our previous observation in normal human astrocytes [32] and appears to limit a putative in vivo use of CA for cancer treatment. However, the cooperative activities of CA with Tz in ERBB2^+^ BC cells and the observation that 90 d administration of rosemary extract (equivalent to 20–60 mg/Kg/d of carnosol plus CA) in rats revealed no adverse effects [17] warrant further studies on the possible therapeutic use of CA in ERBB2^+^ BCs.

Currently, the attempt to block the cellular survival functions of autophagy in cancer by combining chemotherapy with the autophagy inhibitor cloroquine and/or hydroxychloroquine is undergoing promising clinical trials in several aggressive tumors [16]. Yet, a possible alternative strategy in fighting resistance or boosting the anticancer activity of specific antibodies is to couple the targeting of ERBB2 with phytochemical compounds with low toxicity profiles, such as CA [17], which may interfere with therapy-induced protective autophagy as standalone drugs.

## Conclusions

Our study shows for the first time that CA and Tz cooperate in inhibiting BC cell migration and survival. This latter inhibitory effect, which occurs in both Tz-sensitive and Tz-resistant cells, results from multiple mechanisms that ultimately cause cell cycle arrest and inhibition of the late stages of the autophagic flux (Fig. 8c). Given the increasing efforts to find pharmacologically safe natural compounds with anticancer properties, our results warrant further in vivo studies to establish the full potential of CA or CA analogues as adjuvant drugs in the treatment of ERBB2^+^ cancers.

## Additional files

Additional file 1: Table S1. Antibodies used for immunoblot analysis. (DOCX 78 kb)

Additional file 2: Figure S2. CA inhibits ERBB2^−^ in vitro breast cancer cell survival and migration. MCF7 and MDA-MB-231 cells were cultured for 10 days with control medium (Ctr), CA, Tz or CA + Tz supplemented medium, which was changed every 48 h. **a** Cell survival is expressed as arbitrary units (A.U.) after exposure to MTT agent for 4 h. Polynomial (Poly.) interpolation curves are shown. Mean values and standard deviation (indicated as vertical bars) from three independent replicates are shown. **b** MDA-MB-231 cells were cultured after overnight starvation for 7d with control medium (Ctr) or CA supplemented medium in the lower migration chamber, which was changed every 48 h. Representative images of the lower side of the migration membranes stained with Crystal Violet. Bar = 100 μm. **c** Relative cell migration expressed as percentage (%) compared to control was determined as detailed in Methods section. Mean values and standard deviation (indicated as vertical bars) from four independent replicates (*n* = 4) are shown. *P* < 0.001 (***) (TIFF 10150 kb)

Additional file 3: Figure S3. CA and Tz cooperative inhibition of ERBB2^+^ cell survival is transient. Blue lines represent cell survival of SKBR-3 and BT474 cells continuously cultured with CA + Tz (cultures #2). Red lines represent cell survival of SKBR-3 and BT474 cells cultured for up to 6d with CA + Tz and for further 8d with control medium (cultures #1). Cell survival is expressed as arbitrary units (A.U.) after exposure of cultures to Alamar Blue for 4 h. The arrow represents the time point at which the medium containing the two drugs was replaced with control medium in cultures #1. Mean values and standard deviation (indicated as vertical bars) from four independent replicates (*n* = 4) are shown. *P* < 0.001 (***) (TIFF 817 kb)

Additional file 4: Figure S4. CA impairs survival, increases P21^WAF1^ and p62 expression levels and deranges the lysosomal compartment of MCF10A cells. Cells were cultured for 7d with control medium (Ctr), CA, Tz or CA + Tz supplemented medium, which was changed every 48 h. (A) Cell survival is expressed as arbitrary units (A.U.) after exposure to MTT agent for 4 h. Mean values and standard deviation (indicated as vertical bars) from three independent replicates (*n* = 3) are shown. *P* < 0.05 (*), *P* < 0.01 (**), *P* < 0.001 (***). (B) A representative immunoblot analysis is shown, which was performed with antibodies specific for P27^KIP1^, P21^WAF1^, p62 and Tubulin on whole cell lysates. Tubulin is shown as loading controls. Numbers on each lane represent protein levels determined as described in Methods. (C) Representative confocal images of SKBR-3 cells cultured for 7d with CA, Tz and CA + Tz and untreated cells. Cells were fixed, permeabilized and challenged with anti-LAMP1 and anti-LAMP2 antibodies to detect lysosomes and Alexa488-conjugated anti-mouse secondary antibody (green signal). Nuclei were stained with DAPI (blue signal). Arrows indicates representative lysosomes in each condition. Of note, in CA and CA + Tz treated cells lysosomes (LAMP1 and LAMP2 positive) appear larger and clustered compared to untreated cells (arrows). Bar = 20 μm (TIFF 4806 kb)

Additional file 5: Figure S5. CA and Tz alone or together do not modify substantially the extent of apoptosis or necrosis in ERBB2^+^ cells. A) FCM analysis of SKBR-3 and BT474 cells after 48 h of exposure to CA or DMSO. Cells were labeled with sytox blue, allophycocyanin (APC)-conjugated annexin V and resorufin. Both floating and adherent cells were collected for the analysis. The percentage of early apoptotic cells (sytox blue negative, APC-annexin V positive, and resazurine positive), late apoptotic cells (sytox blue positive, APC-annexin V positive) and necrotic cells (sytox blue positive, APC-annexin V negative, and resazurine negative) is shown. Mean values and standard deviation (indicated as vertical bars) from three independent experiments are shown. B) Immunoblot analysis of activated Caspase-9 (Act. Caspase 9) and Tubulin, which is shown as a loading control, in SKBR-3 and BT474 treated with CA and Tz. (TIFF 560 kb)

Additional file 6: Figure S6. Representative DNA content histograms obtained by high resolution DNA flow cytometry from SKBR-3 and BT474 cells, top and bottom row respectively. X axes show DNA content measured as intensity of fluorescent light emitted by DNA bound DAPI at 435 nm; Y axes show number of nuclei (counts). Arrow heads indicate G0/G1 and G2/M peaks. Control treatment, Ctr; Carnosic acid treatment; CA; Trastuzumab treatment, Tz; Carnosic plus Trastuzumab treatment, CA + Tz. (TIFF 396 kb)

Additional file 7: Figure S7. Ki-67 expression modulation by CA and Tz. Cells were cultured for 7d with control medium (Ctr), CA, Tz or CA + Tz supplemented medium, which was changed every 48 h. Cells were fixed and permeabilized and Ki-67 was detected by indirect immunofluorescence analysis using a mouse anti-Ki-67 antibody (see Additional file 1: Table S1) and Alexa488-conjugated goat anti-mouse antibodies. Nuclei were stained with DAPI. Ki-67 positive nuclei and total nuclei were counted in blind. Four microscopic fields (*n* = 4) were analyzed for each experimental condition. Mean values and standard deviation (indicated as vertical bars) are shown. *P* < 0.05 (*), *P* < 0.01 (**), *P* < 0.001 (***). (TIFF 229 kb)

Additional file 8: Figure S8. Analysis of endoplasmic reticulum and ROS stress markers expression. Cells were cultured for 7d with control medium (Ctr), CA, Tz or CA + Tz supplemented medium, which was changed every 48 h. A representative immunoblot analysis is shown, which was performed with anti-HSP70, anti-Catalase and anti-Calreticulin antibodies on whole cell lysates. Tubulin and Actin are shown as loading controls. Numbers on each lane represent protein levels determined as described in Methods. (TIFF 200 kb)

Additional file 9: Figure S9. CA and Tz up-regulate p21^WAF1^ expression levels in SKBR-3 and BT474 cells. Cells were cultured for 7d with control medium (Ctr), CA, Tz or CA + Tz supplemented medium, which was changed every 48 h. A representative immunoblot analysis is shown, which was performed with an anti-p21^WAF1^ antibody on whole cell lysates. Tubulin is shown as loading control. Numbers on each lane represent p21WAF1 protein levels determined as described in Methods. (TIFF 222 kb)

Additional file 10: Figure S10. Analysis of adipogenic markers expression in ERBB2^+^ BC cells. Cells were cultured for 7d with control medium (Ctr), CA, Tz or CA + Tz supplemented medium, which was changed every 48 h. A) PLIN1 and PLIN2 mRNA expression levels were detect by RT qPCR analysis and are expressed as arbitrary units (A.U.). Mean values and standard deviation (indicated as vertical bars) from three independent replicates (*n* = 3) are shown. *P* < 0.05 (*), *P* < 0.01 (**). B) A representative immunoblot analysis is shown, which was performed with an anti-PPARγ antibody on whole cell lysates. Tubulin is shown as loading control. Numbers on each lane represent PPARγ protein levels determined as described in Methods. (TIFF 372 kb)

## Abbreviations

ADC: Antibody-drug conjugate
AKT: RAC-alpha serine/threonine-protein kinase
AL: Autolysosomes
ANOVA: Analysis of variance
ATCC: American Type Culture Collection
ATG5: Autophagy related 5
AV: Autophagic vesicle
BC: Breast cancer
BCL: B-cell lymphoma
CA: Carnosic acid
CDK: Cyclin-dependent kinase
DAPI: 4′,6-Diamidino-2-phenylindole
DMEM: Dulbecco’s modified Eagle’s medium
DMSO: Dimethyl sulfoxide
EGF: Epidermal growth factor
ER: Estrogen receptor
ERBB2^+^: ERBB2 overexpressing
ERK: Extracellular signal regulated kinase
FCM: Flow cytometry
GAPDH: Glyceraldehyde 3-phosphate dehydrogenase
GBM: Glioblastoma
GFAP: Glial fibrillary acid protein
HPLC: High performance liquid chromatography
Hr DNA-FCM: High resolution DNA flow cytometry
HSP: Heat shock protein
IgG: Immunoglobulin G
IKK: IkB kinase
LAMP: Lysosome-associated membrane protein
LC3: Microtubule-associated proteins 1A/1B light chain 3B
LD: Lipid droplets
mTOR: Mammalian target of rapamycin
NF-kB: Nuclear factor kappa-light-chain-enhancer of activated B cells
MAPK: Mitogen-activated protein kinase
MTT: 3-(4,5-dimethylthiazol-2yl)-2,5-diphenyltetrazolium bromide
MVB: Multivesicular body
PFA: Paraformaldehyde
PI3K: Phosphatidyl inositol-3 kinase
PLIN: Perilipin
PPAR: Peroxisome proliferator activated receptor
qPCR: Quantitative polymerase chain reaction
RAS: Rat sarcoma
RAF: Rapidly accelerated fibrosarcoma
ROS: Reactive oxygen species
RT: Real time
SOX2: Sex determining region Y-box 2
TEM: Transmission electron microscopy
Tz: Trastuzumab

## References

[1] Hynes NE, MacDonald G (2009). ErbB receptors and signaling pathways in cancer. Curr Opin Cell Biol.

[2] Nahta R, Esteva FJ (2006). HER2 therapy: molecular mechanisms of trastuzumab resistance. Breast Cancer Res.

[3] Kennecke H, Yerushalmi R, Woods R, Cheang MC, Voduc D, Speers CH (2010). Metastatic behavior of breast cancer subtypes. J Clin Oncol.

[4] Carter P, Presta L, Gorman CM, Ridgway JB, Henner D, Wong WL (1992). Humanization of an anti-p185HER2 antibody for human cancer therapy. Proc Natl Acad Sci U S A.

[5] Vu T, Claret FX (2012). Trastuzumab: updated mechanisms of action and resistance in breast cancer. Front Oncol.

[6] Junttila TT, Akita RW, Parsons K, Fields C, Lewis Phillips GD, Friedman LS (2009). Ligand-independent HER2/HER3/PI3K complex is disrupted by trastuzumab and is effectively inhibited by the PI3K inhibitor GDC-0941. Cancer Cell.

[7] Yakes FM, Chinratanalab W, Ritter CA, King W, Seelig S, Arteaga CL (2002). Herceptin-induced inhibition of phosphatidylinositol-3 kinase and Akt is required for antibody-mediated effects on p27, cyclin D1, and antitumor action. Cancer Res.

[8] Albanell J, Codony J, Rovira A, Mellado B, Gascon P (2003). Mechanism of action of anti-HER2 monoclonal antibodies: scientific update on trastuzumab and 2C4. Adv Exp Med Biol.

[9] Nagata Y, Lan KH, Zhou X, Tan M, Esteva FJ, Sahin AA (2004). PTEN activation contributes to tumor inhibition by trastuzumab, and loss of PTEN predicts trastuzumab resistance in patients. Cancer Cell.

[10] Martinez MT, Perez-Fidalgo JA, Martin-Martorell P, Cejalvo JM, Pons V, Bermejo B (2016). Treatment of HER2 positive advanced breast cancer with T-DM1: a review of the literature. Crit Rev Oncol Hematol.

[11] Uppal H, Doudement E, Mahapatra K, Darbonne WC, Bumbaca D, Shen BQ (2015). Potential mechanisms for thrombocytopenia development with trastuzumab emtansine (T-DM1). Clin Cancer Res.

[12] Baron JM, Boster BL, Barnett CM (2015). Ado-trastuzumab emtansine (T-DM1): a novel antibody-drug conjugate for the treatment of HER2-positive metastatic breast cancer. J Oncol Pharm Pract.

[13] Amaravadi RK, Lippincott-Schwartz J, Yin XM, Weiss WA, Takebe N, Timmer W (2011). Principles and current strategies for targeting autophagy for cancer treatment. Clin Cancer Res.

[14] Chen S, Zhu X, Qiao H, Ye M, Lai X, Yu S (2016). Protective autophagy promotes the resistance of HER2-positive breast cancer cells to lapatinib. Tumour Biol.

[15] Vazquez-Martin A, Oliveras-Ferraros C, Menendez JA (2009). Autophagy facilitates the development of breast cancer resistance to the anti-HER2 monoclonal antibody trastuzumab. PLoS One.

[16] Cufi S, Vazquez-Martin A, Oliveras-Ferraros C, Corominas-Faja B, Cuyas E, Lopez-Bonet E (2013). The anti-malarial chloroquine overcomes primary resistance and restores sensitivity to trastuzumab in HER2-positive breast cancer. Sci Rep.

[17] Moore J, Yousef M, Tsiani E. Anticancer Effects of Rosemary (*Rosmarinus officinalis* L.) Extract and Rosemary Extract Polyphenols. Nutrients. 2016;8(11):731. 10.3390/nu8110731 PMC5133115 27869665

[18] Birtic S, Dussort P, Pierre FX, Bily AC, Roller M (2015). Carnosic acid. Phytochemistry.

[19] Baselga-Escudero L, Souza-Mello V, Pascual-Serrano A, Rachid T, Voci A, Demori I, Grasselli E (2017). Beneficial effects of the Mediterranean spices and aromas on non-alcoholic fatty liver disease. Trends Food Sci Technol.

[20] Gao Q, Liu H, Yao Y, Geng L, Zhang X, Jiang L (2015). Carnosic acid induces autophagic cell death through inhibition of the Akt/mTOR pathway in human hepatoma cells. J Appl Toxicol.

[21] Kim YJ, Kim JS, Seo YR, Park JH, Choi MS, Sung MK (2014). Carnosic acid suppresses colon tumor formation in association with antiadipogenic activity. Mol Nutr Food Res.

[22] Kar S, Palit S, Ball WB, Das PK (2012). Carnosic acid modulates Akt/IKK/NF-kappaB signaling by PP2A and induces intrinsic and extrinsic pathway mediated apoptosis in human prostate carcinoma PC-3 cells. Apoptosis.

[23] Cortese K, Daga A, Monticone M, Tavella S, Stefanelli A, Aiello C (2016). Carnosic acid induces proteasomal degradation of Cyclin B1, RB and SOX2 along with cell growth arrest and apoptosis in GBM cells. Phytomedicine.

[24] Einbond LS, Wu HA, Kashiwazaki R, He K, Roller M, Su T (2012). Carnosic acid inhibits the growth of ER-negative human breast cancer cells and synergizes with curcumin. Fitoterapia.

[25] Gonzalez-Vallinas M, Molina S, Vicente G, Sanchez-Martinez R, Vargas T, Garcia-Risco MR (2014). Modulation of estrogen and epidermal growth factor receptors by rosemary extract in breast cancer cells. Electrophoresis.

[26] Bisio A, De Mieri M, Milella L, Schito AM, Parricchi A, Russo D (2017). Antibacterial and hypoglycemic Diterpenoids from salvia Chamaedryoides. J Nat Prod.

[27] Richheimer SL, Bernart MW, King GA, Kent MC, Bailey DT (1996). Antioxidant activity of lipid-soluble phenolic diterpenes from rosemary. J Am Oil Chem Soc.

[28] Denizot F, Lang R (1986). Rapid colorimetric assay for cell growth and survival. Modifications to the tetrazolium dye procedure giving improved sensitivity and reliability. J Immunol Methods.

[29] Schindelin J, Rueden CT, Hiner MC, Eliceiri KW (2015). The ImageJ ecosystem: an open platform for biomedical image analysis. Mol Reprod Dev.

[30] Limame R, Wouters A, Pauwels B, Fransen E, Peeters M, Lardon F (2012). Comparative analysis of dynamic cell viability, migration and invasion assessments by novel real-time technology and classic endpoint assays. PLoS One.

[31] Monticone M, Biollo E, Fabiano A, Fabbi M, Daga A, Romeo F (2009). Z-Leucinyl-leucinyl-norleucinal induces apoptosis of human glioblastoma tumor-initiating cells by proteasome inhibition and mitotic arrest response. Mol Cancer Res.

[32] Cortese K, Sahores M, Madsen CD, Tacchetti C, Blasi F (2008). Clathrin and LRP-1-independent constitutive endocytosis and recycling of uPAR. PLoS One.

[33] Pfaffl MW (2001). A new mathematical model for relative quantification in real-time RT-PCR. Nucleic Acids Res.

[34] Neve RM, Chin K, Fridlyand J, Yeh J, Baehner FL, Fevr T (2006). A collection of breast cancer cell lines for the study of functionally distinct cancer subtypes. Cancer Cell.

[35] Soule HD, Maloney TM, Wolman SR, Peterson WD, Brenz R, CM MG (1990). Isolation and characterization of a spontaneously immortalized human breast epithelial cell line, MCF-10. Cancer Res.

[36] Qu Y, Han B, Yu Y, Yao W, Bose S, Karlan BY (2015). Evaluation of MCF10A as a reliable model for normal human mammary epithelial cells. PLoS One.

[37] Scholzen T, Gerdes J (2000). The Ki-67 protein: from the known and the unknown. J Cell Physiol.

[38] Baselga J, Albanell J, Molina MA, Arribas J (2001). Mechanism of action of trastuzumab and scientific update. Semin Oncol.

[39] Cuello M, Ettenberg SA, Clark AS, Keane MM, Posner RH, Nau MM (2001). Down-regulation of the erbB-2 receptor by trastuzumab (herceptin) enhances tumor necrosis factor-related apoptosis-inducing ligand-mediated apoptosis in breast and ovarian cancer cell lines that overexpress erbB-2. Cancer Res.

[40] Neve RM, Sutterluty H, Pullen N, Lane HA, Daly JM, Krek W (2000). Effects of oncogenic ErbB2 on G1 cell cycle regulators in breast tumour cells. Oncogene.

[41] Le XF, Claret FX, Lammayot A, Tian L, Deshpande D, LaPushin R (2003). The role of cyclin-dependent kinase inhibitor p27Kip1 in anti-HER2 antibody-induced G1 cell cycle arrest and tumor growth inhibition. J Biol Chem.

[42] Alkarain A, Jordan R, Slingerland J (2004). p27 deregulation in breast cancer: prognostic significance and implications for therapy. J Mammary Gland Biol Neoplasia.

[43] Bjorkoy G, Lamark T, Pankiv S, Overvatn A, Brech A, Johansen T (2009). Monitoring autophagic degradation of p62/SQSTM1. Methods Enzymol.

[44] Mehrpour M, Esclatine A, Beau I, Codogno P (2010). Overview of macroautophagy regulation in mammalian cells. Cell Res.

[45] Koay DC, Zerillo C, Narayan M, Harris LN, DiGiovanna MP (2010). Anti-tumor effects of retinoids combined with trastuzumab or tamoxifen in breast cancer cells: induction of apoptosis by retinoid/trastuzumab combinations. Breast Cancer Res.

[46] Bickel PE, Tansey JT, Welte MA (2009). PAT proteins, an ancient family of lipid droplet proteins that regulate cellular lipid stores. Biochim Biophys Acta.

[47] Visanji JM, Thompson DG, Padfield PJ (2006). Induction of G2/M phase cell cycle arrest by carnosol and carnosic acid is associated with alteration of cyclin a and cyclin B1 levels. Cancer Lett.

[48] Rodriguez CE, Reidel SI, Bal de Kier Joffe ED, Jasnis MA, Fiszman GL (2015). Autophagy protects from Trastuzumab-induced Cytotoxicity in HER2 Overexpressing breast tumor spheroids. PLoS One.

[49] Zhang MH, Man HT, Zhao XD, Dong N, Ma SL (2014). Estrogen receptor-positive breast cancer molecular signatures and therapeutic potentials (review). Biomed Rep.

[50] Gaya M, Repetto V, Toneatto J, Anesini C, Piwien-Pilipuk G, Moreno S (2013). Antiadipogenic effect of carnosic acid, a natural compound present in Rosmarinus Officinalis, is exerted through the C/EBPs and PPARgamma pathways at the onset of the differentiation program. Biochim Biophys Acta.

[51] Rau O, Wurglics M, Paulke A, Zitzkowski J, Meindl N, Bock A (2006). Carnosic acid and carnosol, phenolic diterpene compounds of the labiate herbs rosemary and sage, are activators of the human peroxisome proliferator-activated receptor gamma. Planta Med.

[52] Koppen A, Kalkhoven E (2010). Brown vs white adipocytes: the PPARgamma coregulator story. FEBS Lett.

[53] Larigauderie G, Furman C, Jaye M, Lasselin C, Copin C, Fruchart JC (2004). Adipophilin enhances lipid accumulation and prevents lipid efflux from THP-1 macrophages: potential role in atherogenesis. Arterioscler Thromb Vasc Biol.

[54] Kimmel AR, Brasaemle DL, McAndrews-Hill M, Sztalryd C, Londos C (2010). Adoption of PERILIPIN as a unifying nomenclature for the mammalian PAT-family of intracellular lipid storage droplet proteins. J Lipid Res.

[55] Tansey JT, Sztalryd C, Gruia-Gray J, Roush DL, Zee JV, Gavrilova O (2001). Perilipin ablation results in a lean mouse with aberrant adipocyte lipolysis, enhanced leptin production, and resistance to diet-induced obesity. Proc Natl Acad Sci U S A.

[56] Martinez-Botas J, Anderson JB, Tessier D, Lapillonne A, Chang BH, Quast MJ (2000). Absence of perilipin results in leanness and reverses obesity in Lepr(db/db) mice. Nat Genet.

[57] Ito S, Murphy CG, Doubrovina E, Jasin M, Moynahan ME (2016). PARP inhibitors in clinical use induce genomic instability in normal human cells. PLoS One.

[58] Risinger AL, Dybdal-Hargreaves NF, Mooberry SL (2015). Breast cancer cell lines exhibit differential sensitivities to microtubule-targeting drugs independent of doubling time. Anticancer Res.

[59] Stavik B, Tinholt M, Sletten M, Skretting G, Sandset PM, Iversen N (2013). TFPIalpha and TFPIbeta are expressed at the surface of breast cancer cells and inhibit TF-FVIIa activity. J Hematol Oncol.

