# Supplementary material for: Cooperative antitumor activities of carnosic acid and Trastuzumab in ERBB2+ breast cancer cells
Source: J Exp Clin Cancer Res. 2017 Nov 3;36:154. doi: 10.1186/s13046-017-0615-0 (PMC5670707; doi:10.1186/s13046-017-0615-0)
Supplement: Supplementary file 1 — Antibodies used for immunoblot analysis. (DOCX 78 kb) [file 13046_2017_615_MOESM1_ESM.docx]

| **Additional file 1. Antibodies used in this study.** | | |
| --- | --- | --- |
| **Target protein and epitope** | **Source** | **Catalog number** |
| Actin | Sigma-Aldrich | A2066 |
| AKT | Cell Signaling | 9272 |
| ATG5 | Novus Biologicals | NB110-53818 |
| Calreticulin | Thermo Scientific | PA3-900 |
| Caspase 9 (activated isoform, Asp315) | Calbiochem | AP1013 |
| Catalase | Sigma-Aldrich | C0979 |
| ERBB2 | Thermo Scientific | Ab-20 |
| HSP70 | Abcam | ab2787 |
| Ki-67 | Cell Signaling | 9449 |
| LAMP1 | Developmental Studies Hybridoma Bank | H4A3 |
| LAMP2 | Developmental Studies Hybridoma Bank | H5C6 |
| p21^WAF1^ | Cell Signaling | 2946 |
| p27^KIP1^ | Cell Signaling | 3686 |
| phospho-AKT (Ser473) | Cell Signaling | 9271 |
| SOD1 | Abcam | ab13498 |
| SQSTM1/p62 | Cell Signaling | 8763 |
| Tubulin | Sigma-Aldrich | T5168 |
| Vinculin | Sigma-Aldrich | V9131 |
