# Supplementary figures and images for: Cooperative antitumor activities of carnosic acid and Trastuzumab in ERBB2+ breast cancer cells
Source: J Exp Clin Cancer Res. 2017 Nov 3;36:154. doi: 10.1186/s13046-017-0615-0 (PMC5670707; doi:10.1186/s13046-017-0615-0)

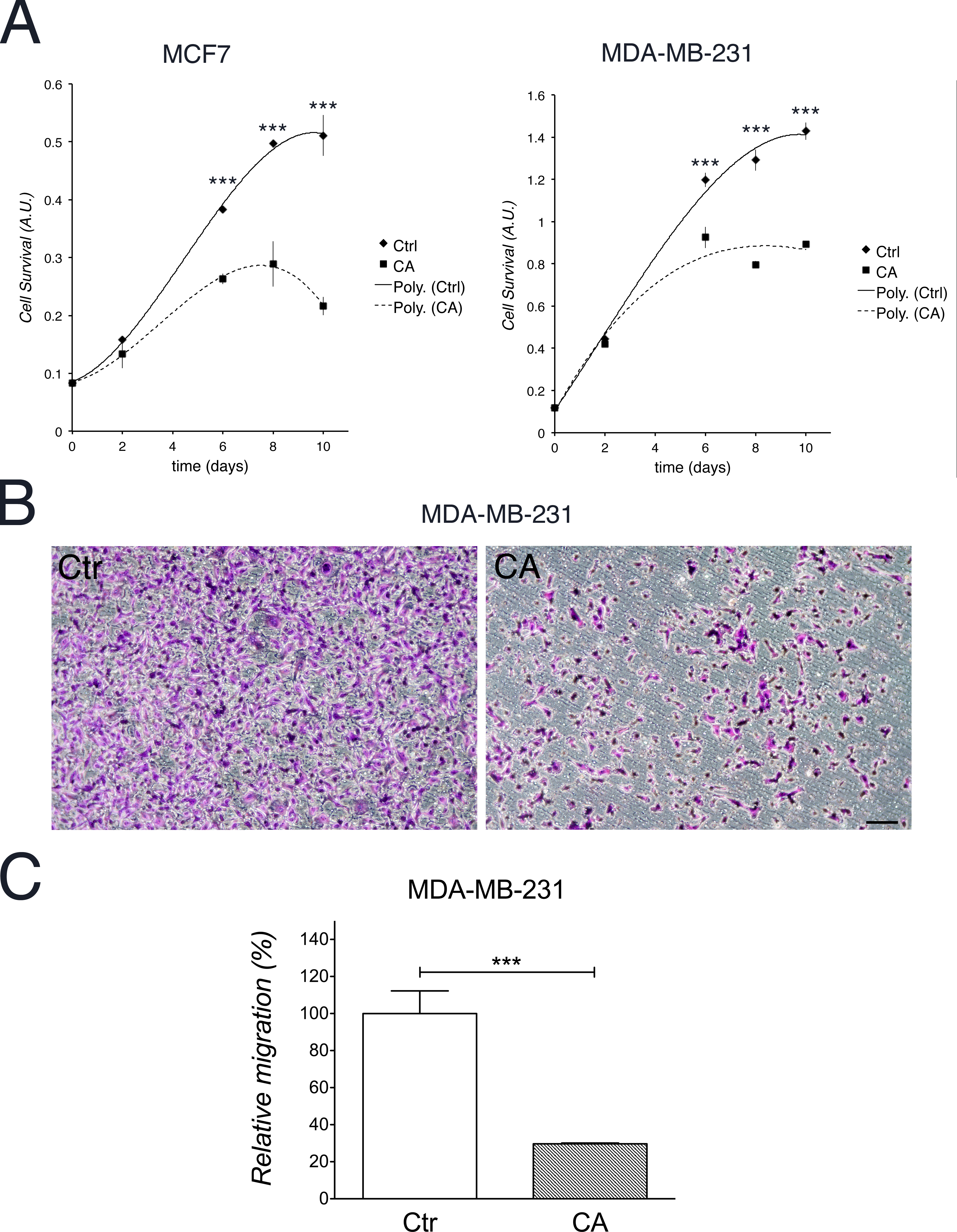

Supplement: Supplementary file 2 — CA inhibits ERBB2− in vitro breast cancer cell survival and migration. MCF7 and MDA-MB-231 cells were cultured for 10 days with control medium (Ctr), CA, Tz or CA + Tz supplemented medium, which was changed every 48 h. a Cell survival is expressed as arbitrary units (A.U.) after exposure to MTT agent for 4 h. Polynomial (Poly.) interpolation curves are shown. Mean values and standard deviation (indicated as vertical bars) from three independent replicates are shown. b MDA-MB-231 cells were cultured after overnight starvation for 7d with control medium (Ctr) or CA supplemented medium in the lower migration chamber, which was changed every 48 h. Representative images of the lower side of the migration membranes stained with Crystal Violet. Bar = 100 μm. c Relative cell migration expressed as percentage (%) compared to control was determined as detailed in Methods section. Mean values and standard deviation (indicated as vertical bars) from four independent replicates (n = 4) are shown. P < 0.001 (***) (TIFF 10150 kb) [file 13046_2017_615_MOESM2_ESM.tif]

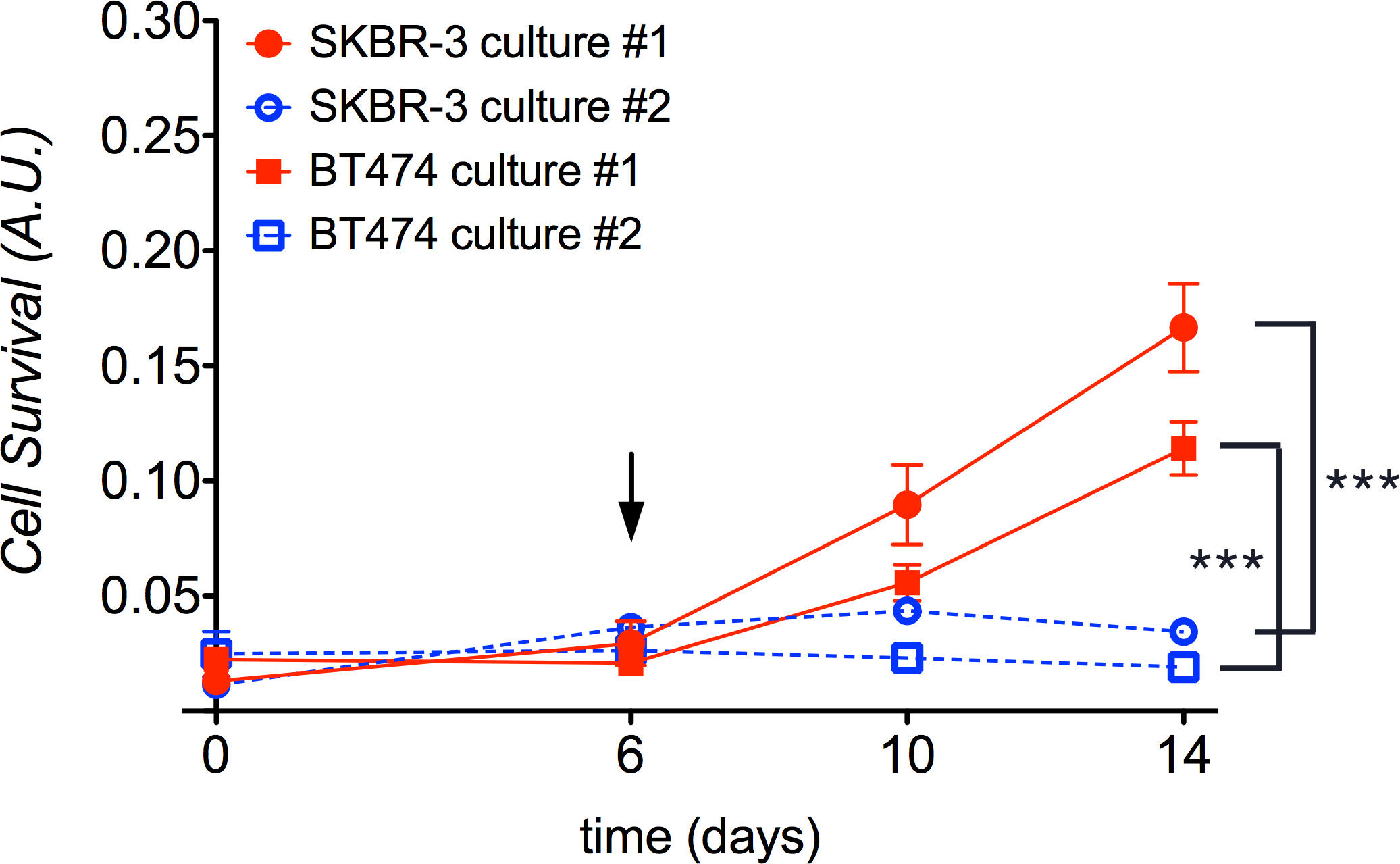

Supplement: Supplementary file 3 — CA and Tz cooperative inhibition of ERBB2+ cell survival is transient. Blue lines represent cell survival of SKBR-3 and BT474 cells continuously cultured with CA + Tz (cultures #2). Red lines represent cell survival of SKBR-3 and BT474 cells cultured for up to 6d with CA + Tz and for further 8d with control medium (cultures #1). Cell survival is expressed as arbitrary units (A.U.) after exposure of cultures to Alamar Blue for 4 h. The arrow represents the time point at which the medium containing the two drugs was replaced with control medium in cultures #1. Mean values and standard deviation (indicated as vertical bars) from four independent replicates (n = 4) are shown. P < 0.001 (***) (TIFF 817 kb) [file 13046_2017_615_MOESM3_ESM.tif]

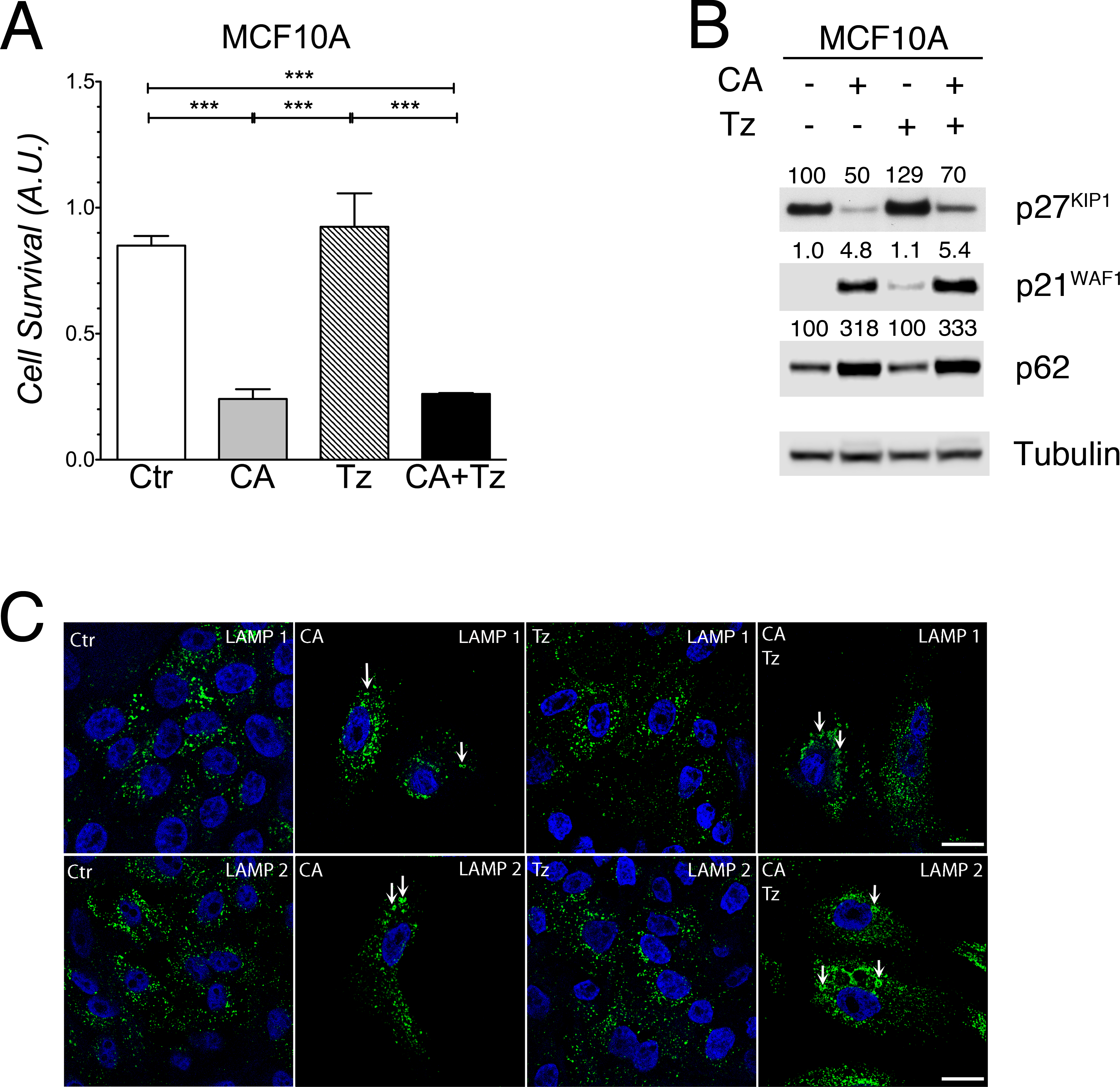

Supplement: Supplementary file 4 — CA impairs survival, increases P21WAF1 and p62 expression levels and deranges the lysosomal compartment of MCF10A cells. Cells were cultured for 7d with control medium (Ctr), CA, Tz or CA + Tz supplemented medium, which was changed every 48 h. (A) Cell survival is expressed as arbitrary units (A.U.) after exposure to MTT agent for 4 h. Mean values and standard deviation (indicated as vertical bars) from three independent replicates (n = 3) are shown. P < 0.05 (*), P < 0.01 (**), P < 0.001 (***). (B) A representative immunoblot analysis is shown, which was performed with antibodies specific for P27KIP1, P21WAF1, p62 and Tubulin on whole cell lysates. Tubulin is shown as loading controls. Numbers on each lane represent protein levels determined as described in Methods. (C) Representative confocal images of SKBR-3 cells cultured for 7d with CA, Tz and CA + Tz and untreated cells. Cells were fixed, permeabilized and challenged with anti-LAMP1 and anti-LAMP2 antibodies to detect lysosomes and Alexa488-conjugated anti-mouse secondary antibody (green signal). Nuclei were stained with DAPI (blue signal). Arrows indicates representative lysosomes in each condition. Of note, in CA and CA + Tz treated cells lysosomes (LAMP1 and LAMP2 positive) appear larger and clustered compared to untreated cells (arrows). Bar = 20 μm (TIFF 4806 kb) [file 13046_2017_615_MOESM4_ESM.tif]

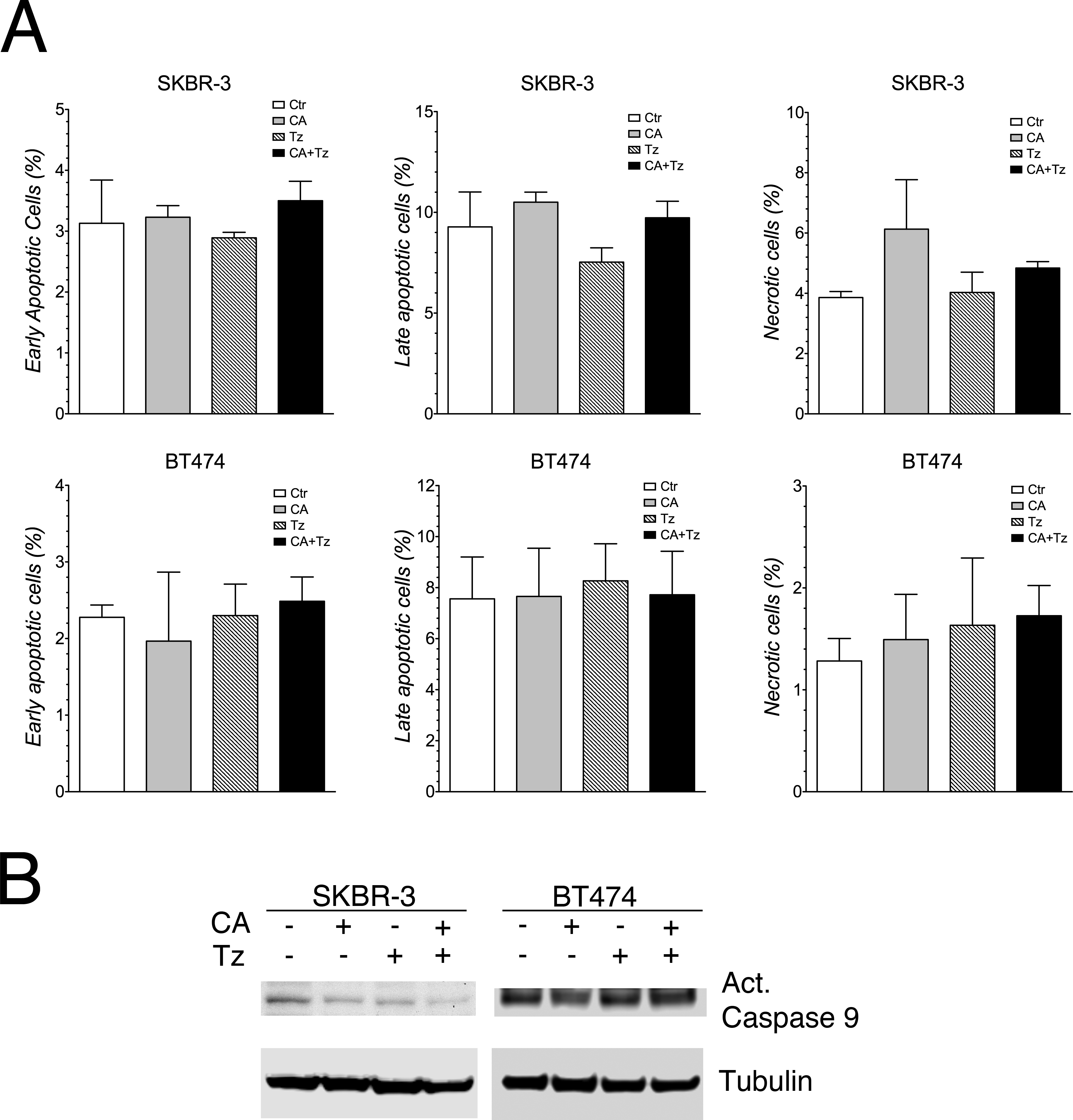

Supplement: Supplementary file 5 — CA and Tz alone or together do not modify substantially the extent of apoptosis or necrosis in ERBB2+ cells. A) FCM analysis of SKBR-3 and BT474 cells after 48 h of exposure to CA or DMSO. Cells were labeled with sytox blue, allophycocyanin (APC)-conjugated annexin V and resorufin. Both floating and adherent cells were collected for the analysis. The percentage of early apoptotic cells (sytox blue negative, APC-annexin V positive, and resazurine positive), late apoptotic cells (sytox blue positive, APC-annexin V positive) and necrotic cells (sytox blue positive, APC-annexin V negative, and resazurine negative) is shown. Mean values and standard deviation (indicated as vertical bars) from three independent experiments are shown. B) Immunoblot analysis of activated Caspase-9 (Act. Caspase 9) and Tubulin, which is shown as a loading control, in SKBR-3 and BT474 treated with CA and Tz. (TIFF 560 kb) [file 13046_2017_615_MOESM5_ESM.tif]

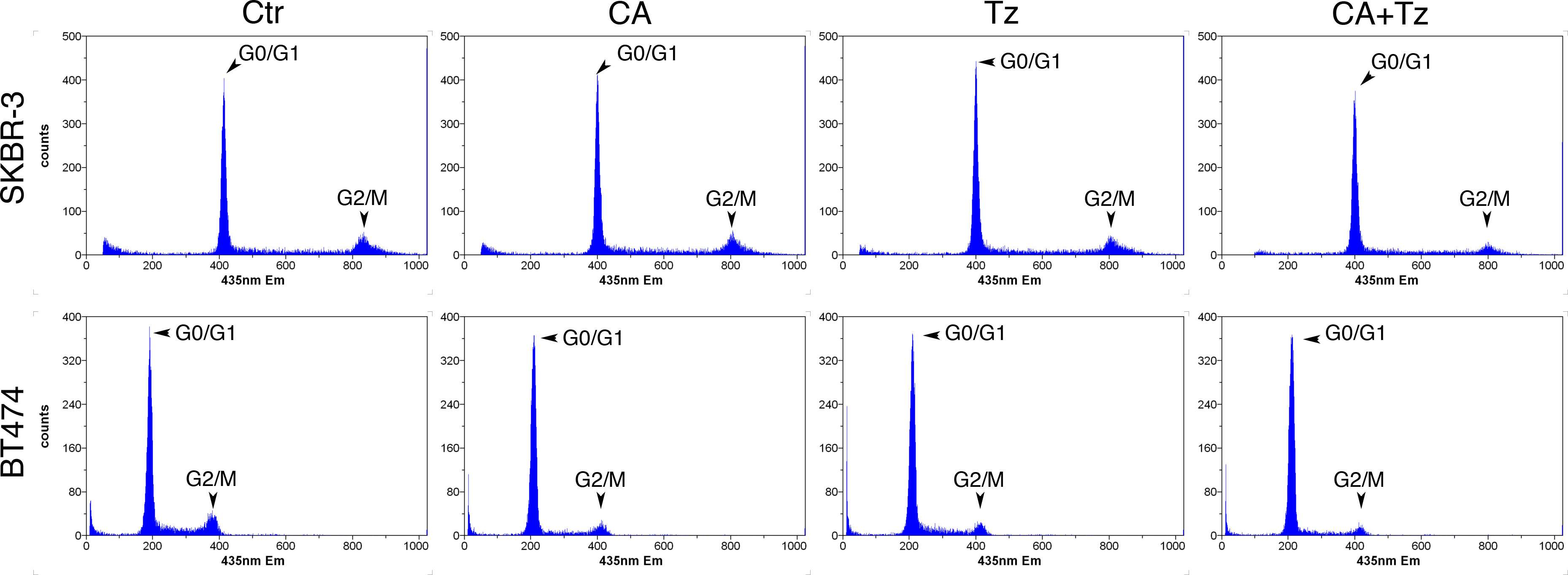

Supplement: Supplementary file 6 — Representative DNA content histograms obtained by high resolution DNA flow cytometry from SKBR-3 and BT474 cells, top and bottom row respectively. X axes show DNA content measured as intensity of fluorescent light emitted by DNA bound DAPI at 435 nm; Y axes show number of nuclei (counts). Arrow heads indicate G0/G1 and G2/M peaks. Control treatment, Ctr; Carnosic acid treatment; CA; Trastuzumab treatment, Tz; Carnosic plus Trastuzumab treatment, CA + Tz. (TIFF 396 kb) [file 13046_2017_615_MOESM6_ESM.tif]

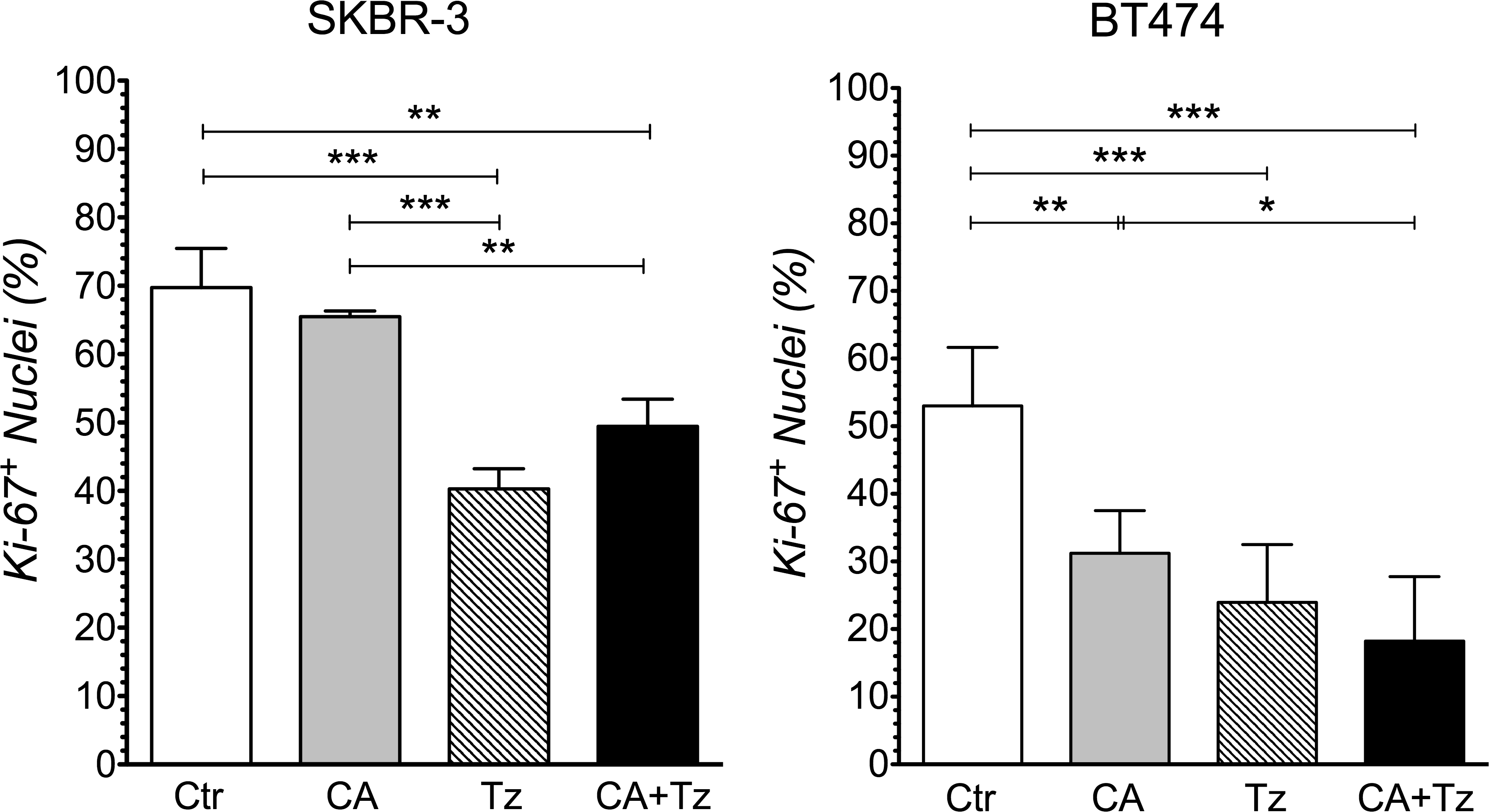

Supplement: Supplementary file 7 — Ki-67 expression modulation by CA and Tz. Cells were cultured for 7d with control medium (Ctr), CA, Tz or CA + Tz supplemented medium, which was changed every 48 h. Cells were fixed and permeabilized and Ki-67 was detected by indirect immunofluorescence analysis using a mouse anti-Ki-67 antibody (see Additional file 1: Table S1) and Alexa488-conjugated goat anti-mouse antibodies. Nuclei were stained with DAPI. Ki-67 positive nuclei and total nuclei were counted in blind. Four microscopic fields (n = 4) were analyzed for each experimental condition. Mean values and standard deviation (indicated as vertical bars) are shown. P < 0.05 (*), P < 0.01 (**), P < 0.001 (***). (TIFF 229 kb) [file 13046_2017_615_MOESM7_ESM.tif]

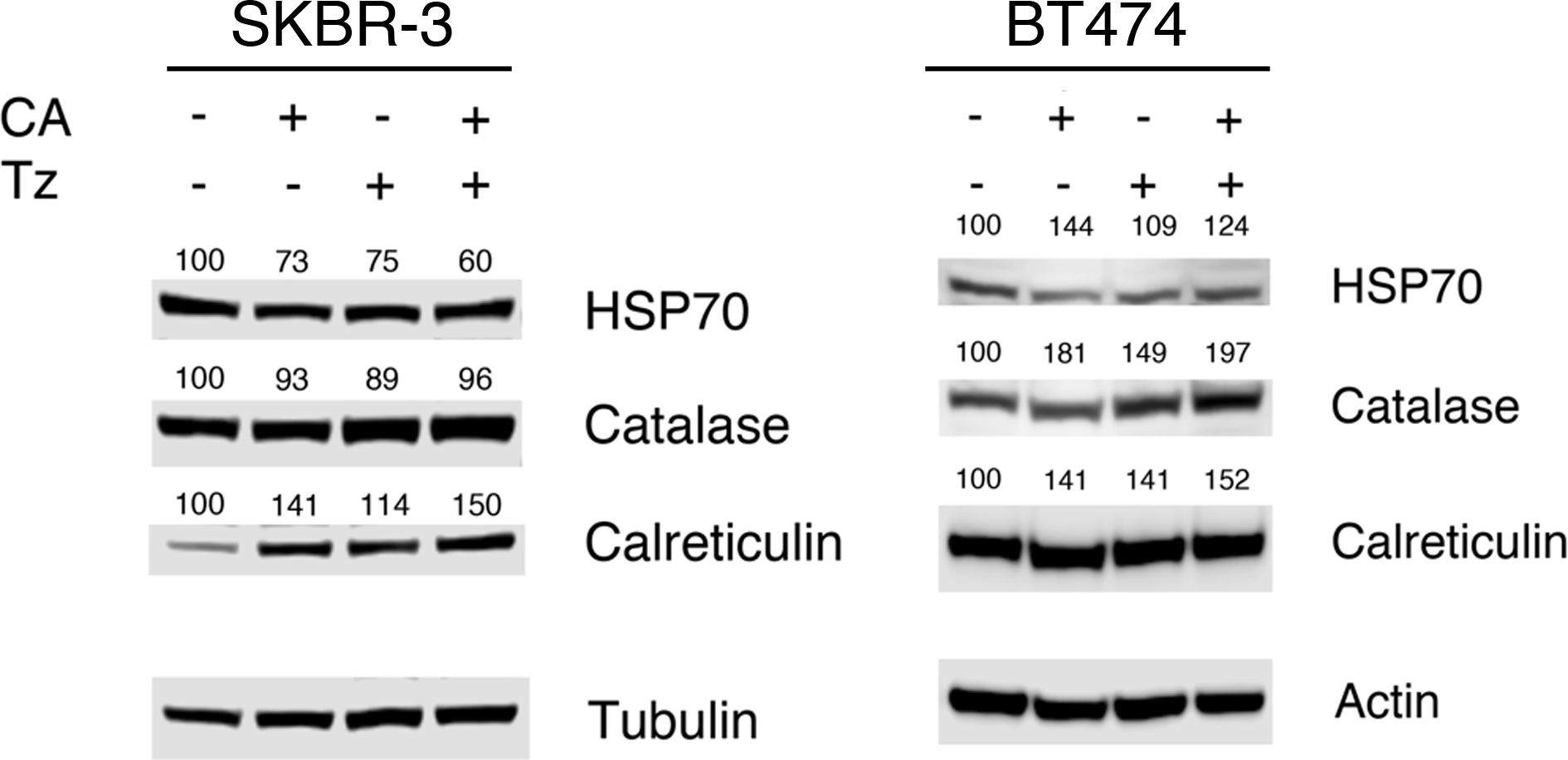

Supplement: Supplementary file 8 — Analysis of endoplasmic reticulum and ROS stress markers expression. Cells were cultured for 7d with control medium (Ctr), CA, Tz or CA + Tz supplemented medium, which was changed every 48 h. A representative immunoblot analysis is shown, which was performed with anti-HSP70, anti-Catalase and anti-Calreticulin antibodies on whole cell lysates. Tubulin and Actin are shown as loading controls. Numbers on each lane represent protein levels determined as described in Methods. (TIFF 200 kb) [file 13046_2017_615_MOESM8_ESM.tif]

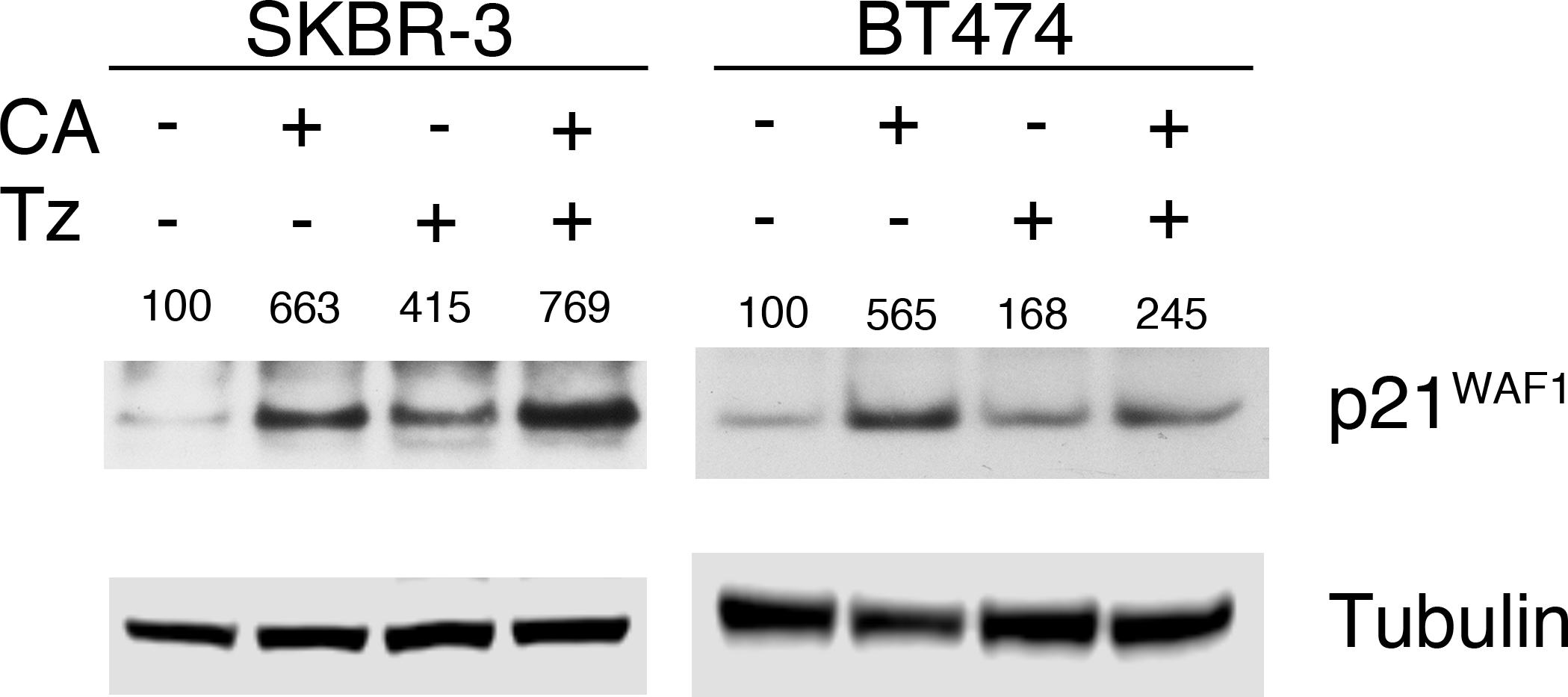

Supplement: Supplementary file 9 — CA and Tz up-regulate p21WAF1 expression levels in SKBR-3 and BT474 cells. Cells were cultured for 7d with control medium (Ctr), CA, Tz or CA + Tz supplemented medium, which was changed every 48 h. A representative immunoblot analysis is shown, which was performed with an anti-p21WAF1 antibody on whole cell lysates. Tubulin is shown as loading control. Numbers on each lane represent p21WAF1 protein levels determined as described in Methods. (TIFF 222 kb) [file 13046_2017_615_MOESM9_ESM.tif]

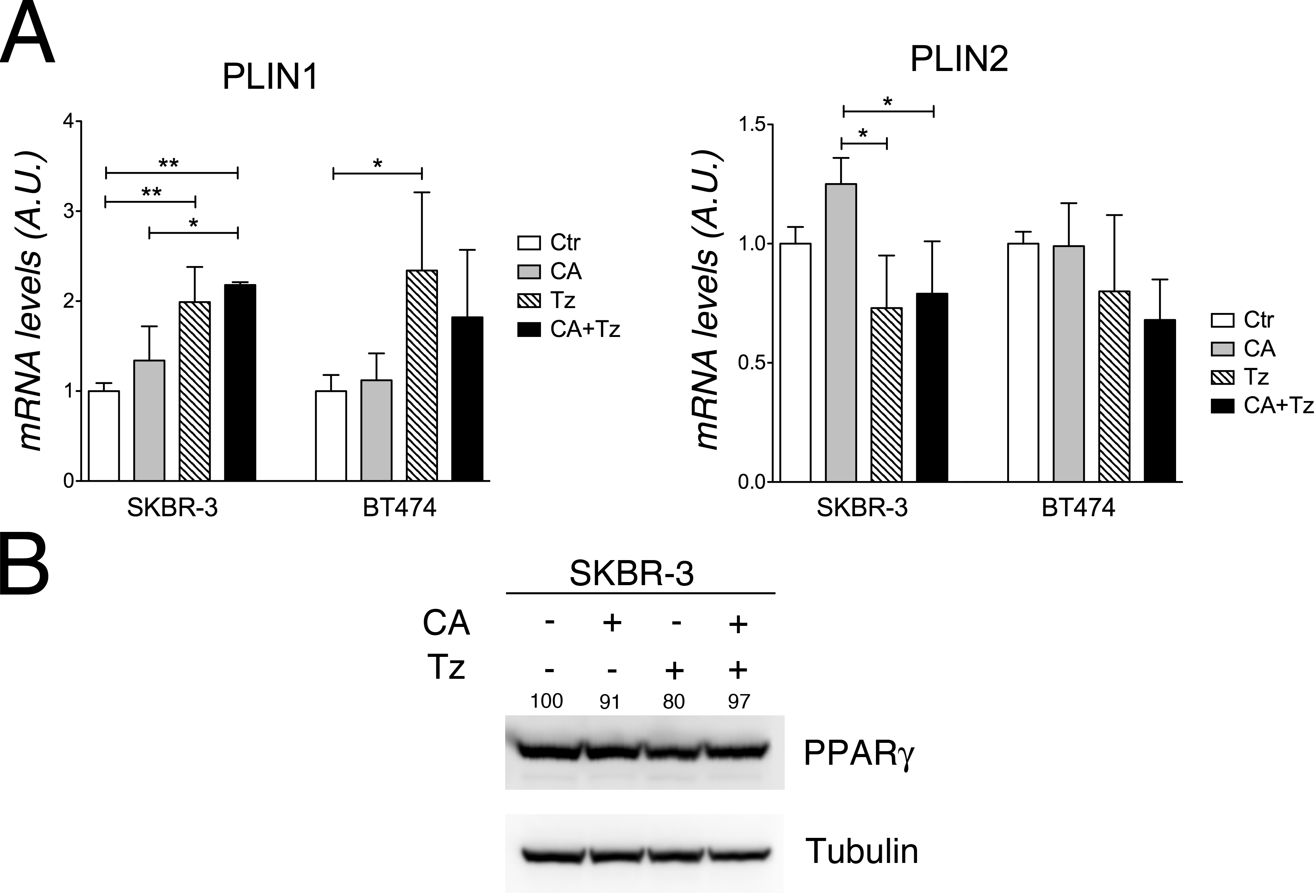

Supplement: Supplementary file 10 — Analysis of adipogenic markers expression in ERBB2+ BC cells. Cells were cultured for 7d with control medium (Ctr), CA, Tz or CA + Tz supplemented medium, which was changed every 48 h. A) PLIN1 and PLIN2 mRNA expression levels were detect by RT qPCR analysis and are expressed as arbitrary units (A.U.). Mean values and standard deviation (indicated as vertical bars) from three independent replicates (n = 3) are shown. P < 0.05 (*), P < 0.01 (**). B) A representative immunoblot analysis is shown, which was performed with an anti-PPARγ antibody on whole cell lysates. Tubulin is shown as loading control. Numbers on each lane represent PPARγ protein levels determined as described in Methods. (TIFF 372 kb) [file 13046_2017_615_MOESM10_ESM.tif]
